# Adipocyte G_q_ signaling is a regulator of glucose and lipid homeostasis in mice

**DOI:** 10.1038/s41467-022-29231-6

**Published:** 2022-03-29

**Authors:** Takefumi Kimura, Sai P. Pydi, Lei Wang, Dhanush Haspula, Yinghong Cui, Huiyan Lu, Gabriele M. König, Evi Kostenis, Gregory R. Steinberg, Oksana Gavrilova, Jürgen Wess

**Affiliations:** 1 Molecular Signaling Section, Laboratory of Bioorganic Chemistry, National Institute of Diabetes and Digestive and Kidney Diseases, Bethesda, MD 20892 USA; 2 Mouse Transgenic Core Facility, National Institute of Diabetes and Digestive and Kidney Diseases, Bethesda, MD 20892 USA; 3 Institute of Pharmaceutical Biology, University of Bonn, 53115 Bonn, Germany; 4 Molecular, Cellular and Pharmacobiology Section, Institute of Pharmaceutical Biology, University of Bonn, 53115 Bonn, Germany; 5 Center for Metabolism, Obesity and Diabetes Research, Department of Medicine and Department of Biochemistry and Biomedical Sciences, McMaster University, Hamilton, ON L8K 4P1 Canada; 6 Mouse Metabolism Core National Institute of Diabetes and Digestive and Kidney Diseases, Bethesda, MD 20892 USA; 7 Present Address: Department of Medicine, Division of Gastroenterology and hepatology, Shinshu University School of Medicine, Matsumoto, 390-8621 Japan

**Keywords:** Metabolism, Medical research, Endocrine system and metabolic diseases, Adipocytes

## Abstract

Obesity is the major driver of the global epidemic in type 2 diabetes (T2D). In individuals with obesity, impaired insulin action leads to increased lipolysis in adipocytes, resulting in elevated plasma free fatty acid (FFA) levels that promote peripheral insulin resistance, a hallmark of T2D. Here we show, by using a combined genetic/biochemical/pharmacologic approach, that increased adipocyte lipolysis can be prevented by selective activation of adipocyte G_q_ signaling in vitro and in vivo (in mice). Activation of this pathway by a G_q_-coupled designer receptor or by an agonist acting on an endogenous adipocyte G_q_-coupled receptor (CysLT_2_ receptor) greatly improved glucose and lipid homeostasis in obese mice or in mice with adipocyte insulin receptor deficiency. Our findings identify adipocyte G_q_ signaling as an essential regulator of whole-body glucose and lipid homeostasis and should inform the development of novel classes of GPCR-based antidiabetic drugs.

## Abstract (web summary)

Obesity impairs regulation of adipocyte lipolysis, which contributes towards development of insulin resistance. Here the authors report that adipocyte Gq signaling functions as a regulator of lipolysis and systemic glucose and lipid homeostasis in mice, suggesting that agents able to stimulate this pathway may prove useful as antidiabetic drugs.

## Introduction

Adipocytes play an important role in the pathogenesis of type 2 diabetes (T2D), and white adipose tissue (WAT) contributes to the regulation of whole-body glucose and energy homeostasis^1–3^. Obesity is characterized by an excess of WAT, associated with an increase in adipocyte size due to increased triglyceride (TG) deposition^4^. Lipolysis in white adipocytes is increased in the obese state, resulting in the release of excessive amounts of free fatty acids (FFAs) into the blood stream^2,4^. This increase in lipolysis is thought to be caused by a decrease in the responsiveness of WAT to the antilipolytic action of insulin^4^. Increased plasma FFA levels lead to the ectopic accumulation of fat in other peripheral tissues involved in the regulation of glucose homeostasis, contributing to peripheral insulin resistance and impaired glucose tolerance^1–3^. In combination with compromised β-cell function, impaired peripheral insulin action eventually leads to the development of T2D^5^. On the basis of these considerations, adipocytes are considered excellent drug targets to improve impaired glucose homeostasis in T2D and related metabolic disorders^2^.

G protein-coupled receptors (GPCRs) are known to regulate key metabolic processes including whole-body glucose and energy homeostasis^6–8^. GPCRs represent a superfamily of cell surface receptors that respond to a large variety of extracellular signals including hormones, neurotransmitters, paracrine factors, and many other agents and stimuli^9,10^. About 30–40% of drugs in current clinical use act on one or more distinct GPCRs, indicative of the extraordinary clinical relevance of this class of receptors^9^.

Like all other cell types, adipocytes express dozens of GPCRs which are coupled to different functional classes of heterotrimeric G proteins^11,12^. Based on the structural and functional properties of the G protein α subunits, heterotrimeric G proteins are subdivided into four major classes: G_s_, G_i/o_, G_q/11_, and G_12/13_^13^. Previous studies have shown that stimulation of adipocyte G_s_ signaling promotes lipolysis, while activation of adipocyte G_i_ signaling exerts antilipolytic effects^11,14–17^. In contrast, little is known about the in vivo metabolic consequences of selectively activating G_q_ signaling in adipocytes.

To address this issue, we used a chemogenetic approach to generate a mouse model that expresses a G_q_-coupled designer GPCR (G_q_ DREADD; G_q_-Coupled Designer Receptor Exclusively Activated by a Designer Drug; alternative names: hM3Dq or simply GqD)^18,19^ selectively in adipocytes (adipo-GqD mice). Treatment of adipo-GqD mice with clozapine-N-oxide (CNO), a synthetic compound that can activate GqD and related DREADDs but is otherwise pharmacologically inert when used in the proper dose/concentration range^18,19^, selectively stimulated G_q_ signaling in adipocytes. We also generated and analyzed several additional mutant mouse models to explore the cellular and molecular mechanisms underlying the metabolic changes caused by the activation of adipocyte G_q_ signaling.

Here, we show that activation of adipocyte G_q_ signaling improves glucose homeostasis and peripheral insulin sensitivity under both physiological and pathophysiological conditions. In vitro and in vivo studies demonstrated that agents that activate adipocyte G_q_ signaling inhibit lipolysis and stimulate glucose uptake in an insulin-independent fashion in both human and mouse adipocytes, leading to reduced plasma FFA levels and improved glucose tolerance. We also demonstrated that activation of a G_q_-coupled receptor (CysLT_2_ receptor) that is endogenously expressed by both mouse and human adipocytes mimics the beneficial metabolic effects observed with CNO-treated adipo-GqD mice. Our findings indicate that adipocyte G_q_ signaling represents a key regulator of whole-body lipid and glucose homeostasis. The identification of drugs able to selectively activate G_q_-coupled receptors endogenously expressed by adipocytes may lead to novel therapies for the management of T2D.

## Results

### Selective activation of adipocyte G_q_ signaling in vivo improves glucose homeostasis

**Fig. 1:**
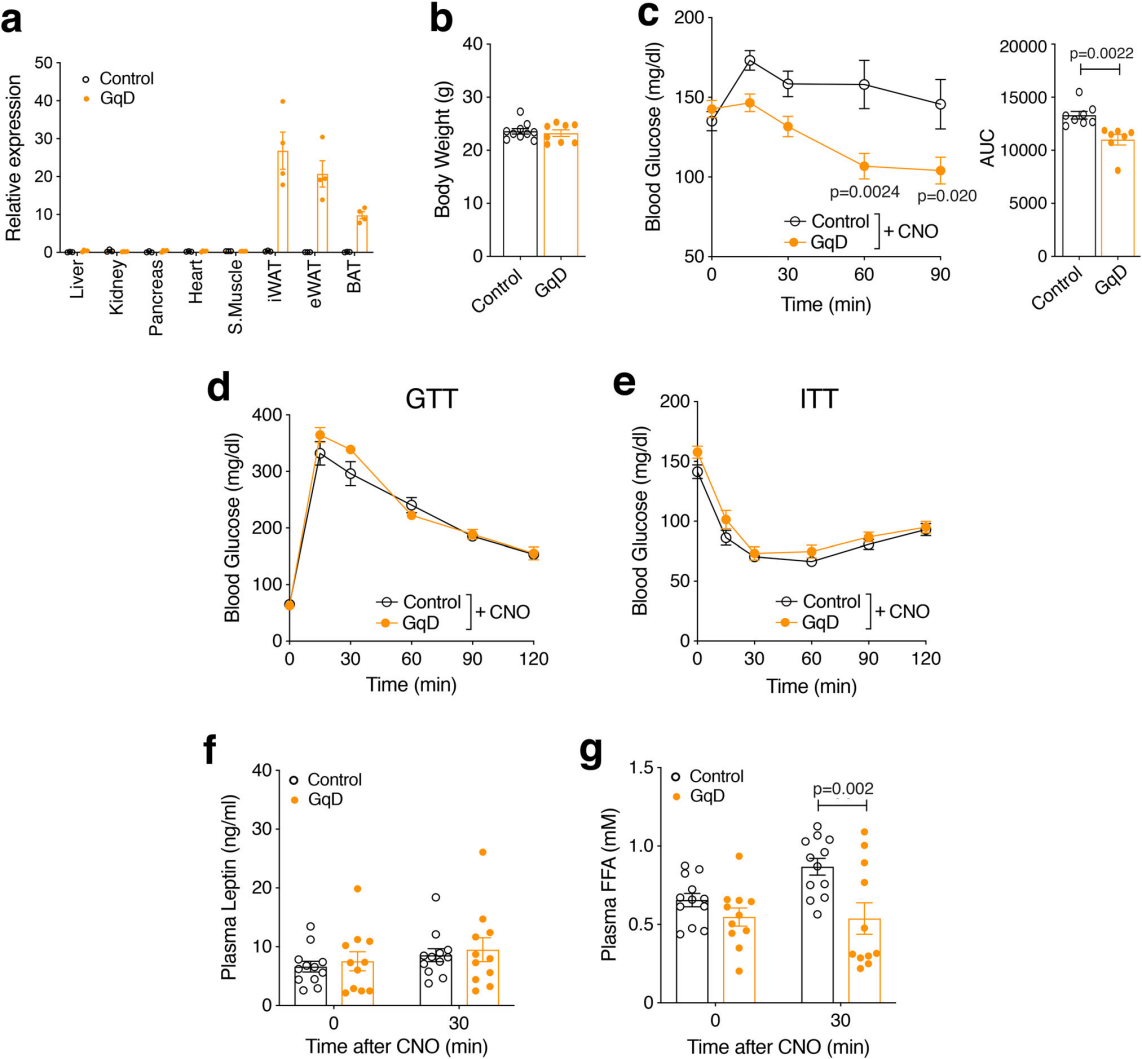
Acute CNO treatment of adipo-GqD mice causes reduced blood glucose and plasma free acid levels. **a** Expression levels of GqD mRNA in different tissues of adipo-GqD (GqD) and control mice (*LSL-hM3Dq* mice that did not harbor the *adipoq-Cre* transgene) (*n* = 4 per group). The presence of GqD transcripts was detected via qRT-PCR. S. Muscle, skeletal muscle; iWAT, inguinal white adipose tissue; eWAT, epididymal white adipose tissue; BAT, brown adipose tissue. **b** Body weights of adipo-GqD and control mice (mouse age: 12 weeks; *n* = 8–10 per group). **c** Blood glucose levels after CNO injection (10 mg/kg i.p.) following a 4 hr fast (*n* = 7–9 per group). **d**, **e** Effect of acute CNO treatment (10 mg/kg i.p.) on glucose tolerance (**d**, GTT) and insulin tolerance (**e**, ITT). GTT: 2 g glucose/kg after an overnight fast; ITT: 0.75 U insulin/kg after a 4 hr fast (*n* = 8 or 9 per group). **f**, **g**, Plasma leptin (**f**), and plasma free fatty acid (FFA) (**g**) levels of control and adipo-GqD mice 30 min after injection of CNO (10 mg/kg i.p.) following a 4 hr fast (*n* = 11 or 12 per group). Metabolic tests (**c**–**g**) were carried out with male mice that were 10-16 weeks old. Data are presented as means ± s.e.m. **b**, **c** (AUC), **f**, **g**: two tailed Student’s *t-t*est; **c**–**e**: two-way ANOVA followed by Bonferroni’s post hoc test). Source data are provided as a Source data file.

To be able to selectively activate G_q_ signaling in adipocytes in vivo, we used a CNO-sensitive designer GPCR that selectivity couples to G proteins of the G_q_ family as a tool (official name: hM3Dq; alternative names: G_q_ DREADD or simply GqD)^18,19^. To generate mutant mice that express the GqD receptor selectively in adipocytes (adipo-GqD mice), we crossed *LSL-hM3Dq* mice^20^ with *adipoq-Cre* mice^21^. Adipo-GqD mice carried one copy of the *LSL-GqD* allele and one copy of the *adipoq-Cre* transgene. *LSL-hM3Dq* mice that did not harbor the *adipoq-Cre* transgene served as control animals throughout all experiments. qRT-PCR studies confirmed that GqD was selectively expressed in adipose tissues of adipo-GqD mice (Fig. 1a).

Initially, we carried out metabolic studies with adipo-GqD mice and their control littermates (males that were at least 8 weeks old) maintained on regular chow diet. Bodyweight did not differ significantly between the two groups of mice (Fig. 1b). Acute CNO treatment (10 mg/kg i.p) led to a pronounced reduction in blood glucose levels in adipo-GqD mice, but not in control littermates (Fig. 1c). In both groups of mice, CNO administration had no significant effect on blood glucose excursions in glucose and insulin tolerance tests (glucose dose: 2 g/kg i.p.; insulin dose: 0.75 U/kg i.p.) (Fig. 1d, e). While CNO treatment did not affect plasma leptin levels in both cohorts of mice (Fig. 1f), CNO caused a significant reduction in plasma FFA levels in adipo-GqD mice (Fig. 1g), indicating that activation of adipocyte G_q_ signaling inhibits lipolysis. Basal plasma insulin levels after a 4 hr fast did not differ significantly between control and adipo-GqD mice (0.58 ± 0.07 ng/ml and 0.44 ± 0.06 ng/ml, respectively) (means ± s.e.m., *n* = 10 per group). CNO treatment (10 mg/kg i.p) of adipo-GqD mice had no significant effect on plasma insulin levels (0.42 ± 0.07 ng/ml; 30 min post-injection).

Treatment of adipo-GqD mice with a lower dose of CNO (3 mg/kg i.p.) or a very low dose (10 μg/kg i.p.) of another DREADD agonist, deschloroclozapine (DCZ)^22^, led to comparable decreases in blood glucose and plasma FFA levels (Supplementary Fig. 1). This observation indicated that the metabolic phenotypes observed after treatment of mice with 10 mg/kg of CNO were not caused by any unspecific effects under our experimental conditions. CNO-injected control mice showed a trend towards increased plasma FFA levels, most likely as a consequence of the injection stress (Supplementary Fig. 1b). In agreement with this notion, injection of control and adipo-GqD mice with saline caused comparable increases in plasma FFA levels (Supplementary Fig. 1c). However, these responses failed to reach statistical significance.

**Fig. 2:**
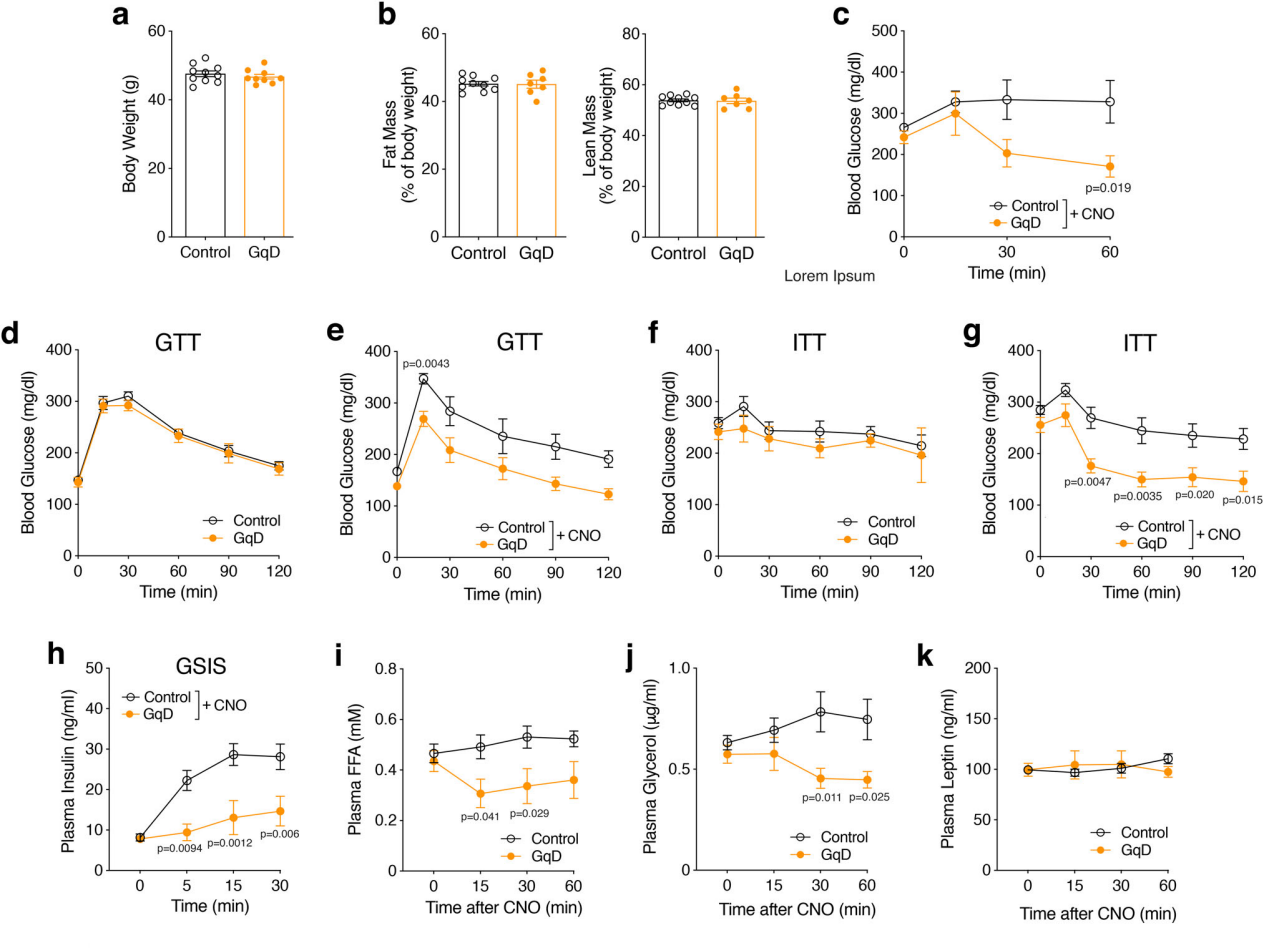
Acute CNO treatment of adipo-GqD mice maintained on an obesogenic diet improves glucose homeostasis and suppresses lipolysis. **a, b** Body weight (**a**) and body composition (fat and lean mass as % of body weight) (**b**) of adipo-GqD and control mice maintained on high-fat diet (HFD) for 10 weeks prior to CNO treatment (*n* = 7–10 per group). *LSL-hM3Dq* mice that did not harbor the *adipoq-Cre* transgene served as control animals throughout all experiments. **c** Blood glucose levels of HFD adipo-GqD and control mice after acute treatment with CNO (10 mg/kg i.p.) following a 4 hr fast. **d**, **e** Glucose tolerance test (GTT) without (**d**) and with (**e**) CNO treatment (10 mg/kg i.p.) after an overnight fast (16 hr). **f**, **g** Insulin tolerance test (ITT) without (**f**) and with (**g**) CNO treatment (10 mg/kg i.p.) after a 4 h fast. **h** Glucose-stimulated insulin secretion (GSIS). Plasma insulin levels of HFD adipo-GqD and control mice after i. p. treatment with glucose (2 g/kg) plus CNO (10 mg/kg) after a 4 h fast. **i**–**k** Plasma FFA (**i**), glycerol (**j**) and leptin (**k**) levels of HFD control and adipo-GqD mice after acute treatment with CNO (10 mg/kg i.p.) following a 4 h fast. In (**c**–**k**) mice were maintained on HFD for 10–14 weeks (*n* = 5–10 per group). Data are shown as means ± s.e.m. **c**–**k**: two-way ANOVA followed by Bonferroni’s post hoc test). Source data are provided as a Source data file.

We next carried out a similar series of experiments using adipo-GqD mice and control littermates (males) maintained on a high-fat diet (HFD) for at least 8 weeks. In male C57BL/6 mice, the genetic background on which the adipo-GqD mice were maintained, this obesogenic diet causes glucose intolerance, hyperglycemia, and insulin resistance, which are all key features of T2D^23,24^. Bodyweight and body composition (lean and fat body mass) did not differ significantly between HFD adipo-GqD and control mice maintained on HFD for 10 weeks (Fig. 2a, b). While acute CNO treatment (10 mg/kg i.p.) of HFD control mice had little effect on blood glucose levels, CNO-treated HFD adipo-GqD mice displayed a striking reduction in blood glucose levels (Fig. 2c).

In the absence of co-injected CNO, HFD adipo-GqD and control mice showed comparable impairments in glucose tolerance and insulin sensitivity (Fig. 2d, f). In contrast, following co-injection of glucose (1 g/kg i.p.) or insulin (1 U/kg i.p.) with CNO (10 mg/kg i.p.), HFD adipo-GqD mice displayed significant improvements in glucose tolerance and insulin sensitivity, respectively (Fig. 2e, g). Following co-injection of glucose (1 g/kg i.p.) with CNO, glucose-stimulated insulin secretion (GSIS) was significantly reduced in HFD adipo-GqD mice, as compared to the corresponding control animals (Fig. 2h), most likely as a result of improved glucose homeostasis caused by adipocyte G_q_ activation. In agreement with the plasma FFA data obtained with mice maintained on regular chow (Fig. 1g), CNO treatment caused a significant reduction in plasma FFA and glycerol levels in HFD adipo-GqD mice (Fig. 2i, j). This CNO effect was not observed with HFD control littermates (Fig. 2i, j), confirming that activation of adipocyte G_q_ signaling inhibits lipolysis in vivo. CNO treatment had no significant effect on plasma leptin levels in both groups of mice (Fig. 2k).

To explore the metabolic effects of chronic activation of adipocyte G_q_ signaling, we injected adipo-GqD mice and control littermates consuming regular chow daily with CNO (10 mg/kg i.p.) for 11 days (Supplementary Fig. 2). While chronic CNO treatment did not lead to any genotype-dependent changes in body weight and insulin tolerance (Supplementary Fig. 2a, c), CNO-treated adipo-GqD mice showed improved glucose tolerance (Supplementary Fig. 2b). Moreover, following repeated CNO treatment, adipo-GqD mice consistently displayed significantly reduced plasma FFA levels (Supplementary Fig. 2d). In contrast, chronic CNO treatment of control littermates had no significant effect on plasma FFA levels (Supplementary Fig. 2d). These data indicate that chronic activation of adipocyte G_q_ signaling also results in significant metabolic improvements in mice.

### CNO treatment of adipo-GqD mice promotes glucose uptake by inguinal WAT and heart

To examine whether activation of adipocyte G_q_ signaling promoted glucose uptake into adipose tissues, we performed an in vivo ^14^C-2-deoxyglucose (^14^C-2-DG) uptake assay (Supplementary Fig. 3a). Adipo-GqD mice and control littermates were fasted overnight and then injected with CNO (10 mg/kg i.p.) and a trace amount of ^14^C-2-DG. Forty min later, the mice were euthanized, and the ^14^C-2-DG content of inguinal WAT (iWAT), epididymal WAT (eWAT), brown adipose tissue (BAT), various skeletal muscle tissues, heart, and liver was determined. We found that CNO treatment caused a significant increase in stimulated glucose uptake (^14^C-2-DG accumulation) in iWAT and heart of adipo-GqD mice (Supplementary Fig. 3a).

One possible explanation for the increase in cardiac glucose uptake displayed by the adipo-GqD mice is that the CNO-induced lowering of plasma FFA levels resulting from activation of adipocyte G_q_ signaling led to an increased usage of glucose as a cardiac fuel source^25,26^. To further corroborate this concept, we subjected mice that expressed a G_i_-coupled DREADD selectively in adipocytes (adipo-GiD mice)^14^ to ^14^C-2-DG uptake studies. We previously demonstrated that CNO treatment of adipo-GiD mice results in greatly reduced plasma FFA acid levels^14^, similar to our current findings with CNO-treated adipo-GqD mice. In agreement with the data obtained with the adipo-GqD mice (Supplementary Fig. 3a), CNO (10 mg/kg i.p.) treatment of adipo-GiD mice resulted in a significant increase in ^14^C-2-DG uptake in the heart (Supplementary Fig. 3b). Taken together, these data are in agreement with previous studies that lowering of plasma FFA levels promotes the usage of glucose as a cardiac fuel source.

### Functional and biochemical studies with differentiated mouse 3T3F442A adipocytes

To gain insight into the cellular and molecular mechanisms through which activation of adipocyte G_q_ signaling exerts its beneficial metabolic actions in vivo, we carried out studies with cultured, fully differentiated 3T3F442A mouse adipocytes. Infection of 3T3F442A cells with an adenovirus coding for GqD resulted in GqD-expressing 3T3F442A cells (GqD-3T3F442A cells) (Supplementary Fig. 4a). For control purposes, we infected 3T3F442A cells with an adenovirus encoding GFP, resulting in GFP-3T3F442A control cells. In experiments exploring the functional properties of GqD- and GFP-3T3F442A cells, both cell lines were processed in parallel.

**Fig. 3:**
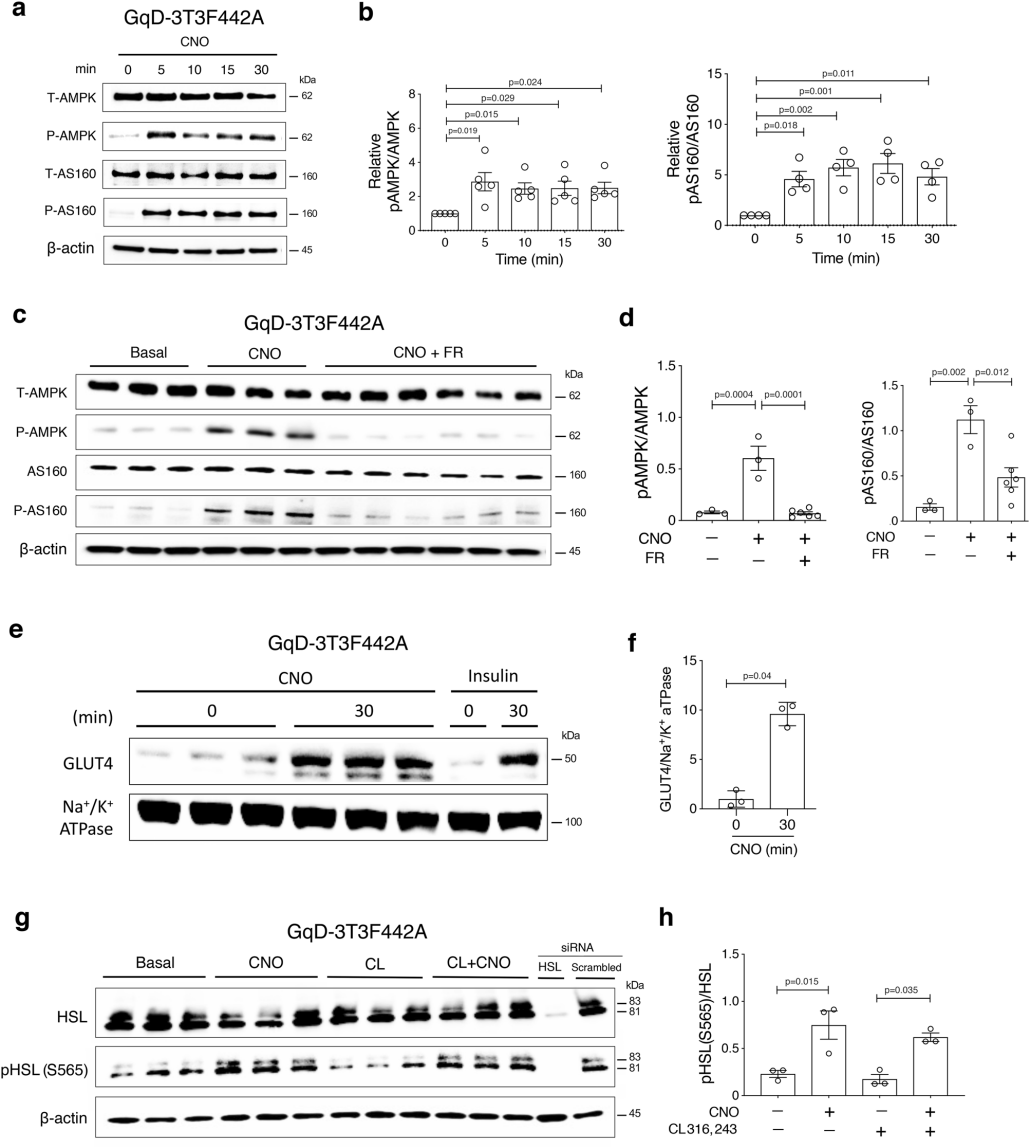
Gq-meditated stimulation of AMPK phosphorylation, GLUT4 translocation, and phosphorylation of HSL (S565) in 3T3F442A mouse adipocytes. **a** Western blotting studies with GqD-expressing mouse 3T3F442A cells (GqD-3T3F442A cells) treated with CNO (10 μM) for different periods of time. **b** Quantitative analysis of pAMPK/AMPK and pAS160/AS160 protein expression levels shown in (**a**). Data shown in (**b**) were derived from four of five independent experiments. Protein expression levels were normalized relative to protein expression levels at time ‘0’. **c** Western blotting studies carried out in the presence of a G_q/11_ inhibitor. GqD-3T3F442A cells) were incubated with CNO (10 μM) for 30 min either in the absence or presence of FR900359 (FR; 1 μM), a G_q/11_ inhibitor. **d** Quantitative analysis of pAMPK/AMPK and pAS160/AS160 protein expression levels shown in (**c**). **e** Immunoblotting analysis of GLUT4 plasma membrane localization. After treatment of GqD-3T3F442A cells with CNO (10 μM) or insulin (10 nM) for 30 min, the plasma membrane fraction was isolated and subjected to immunoblotting analysis using an anti-GLUT4 antibody. Na + /K + ATPase served as a marker for plasma membrane proteins. **f** Quantitative analysis of GLUT4 expression levels shown in (**e**). **g** Western blot analysis of HSL phosphorylation at position S565. GqD-3T3F442A cells were treated with CNO (10 μM), CL316,243 (CL; 100 nM), a β3-adrenergic receptor agonist, or a combination of both drugs. To demonstrate the specificity of the HSL antibody used, GqD-3T3F442A cells were treated with HSL-specific siRNA or scrambled control siRNA. **h** Quantitative analysis of pHSL(S565)/HSL protein expression levels shown in (**g**). Data are presented as means ± s.e.m. **b**, **d**, **f**, **h**: one-way ANOVA followed by Bonferroni’s post-hoc test). Source data are provided as a Source data file.

Western blotting studies demonstrated that CNO (10 μM) treatment of differentiated GqD-3T3F442A cells promoted the phosphorylation of AMPK and AS160 (Tbc1d4) (Fig. 3a, b) (see Supplementary Fig. 5a for the specificity of the AS160 antibody). These effects could be blocked by co-incubation with FR900359 (FR; 1 μM), a selective inhibitor of G_q/11_^27^ (Fig. 3c, d). Cell fractionation studies showed that incubation of GqD-3T3F442A cells with CNO (10 μM) resulted in a pronounced translocation of GLUT4 to the plasma membrane (Fig. 3e, f), a response that is typically observed after activation of AMPK signaling in adipocytes^28^. The magnitude of this response was similar to that of 10 nM insulin. At the same time, CNO treatment of GqD-3T3F442A cells resulted in reduced levels of cytoplasmic GLUT4 (Supplementary Fig. 5b). CNO (10 μM) treatment of GFP-3T3F442A control cells had no significant effect on the phosphorylation status of AMPK or GLUT4 translocation (Supplementary Fig. 4b, c).

Hormone-sensitive lipase (HSL) catalyzes a key step in the breakdown of triglycerides^29^. Previous studies have shown that activated AMPK phosphorylates HSL at S565, resulting in the inhibition of HSL activity^30,31^. In agreement with the observed increase in pAMPK levels (Fig. 3a, b), CNO treatment of GqD-3T3F442A cells stimulated the phosphorylation of HSL at S565 (Fig. 3e, f). In contrast, treatment of GqD-3T3F442A cells with CL316,243 (100 nM), a β3-adrenergic receptor (β3-AR) agonist that leads to G_s_-dependent activation of PKA, had no significant effect on the phosphorylation status of S565 (Fig. 3g, h). However, co-incubation of GqD-3T3F442A cells with CL316,243 and CNO promoted HSL phosphorylation at S565, as compared to treatment with CL316,243 alone (Fig. 3g, h).

To explore whether similar changes occurred in vivo, we injected adipo-GqD mice and control littermates with CNO (10 mg/kg i.p.) and then harvested iWAT 5 min later. Western blotting studies confirmed that CNO-stimulated adipocyte G_q_ signaling promoted the accumulation of pAMPK and pHSL(S565) in iWAT **(**Supplementary Fig. 6). These data agree very well with the in vitro data described in the previous paragraph.

**Fig. 4:**
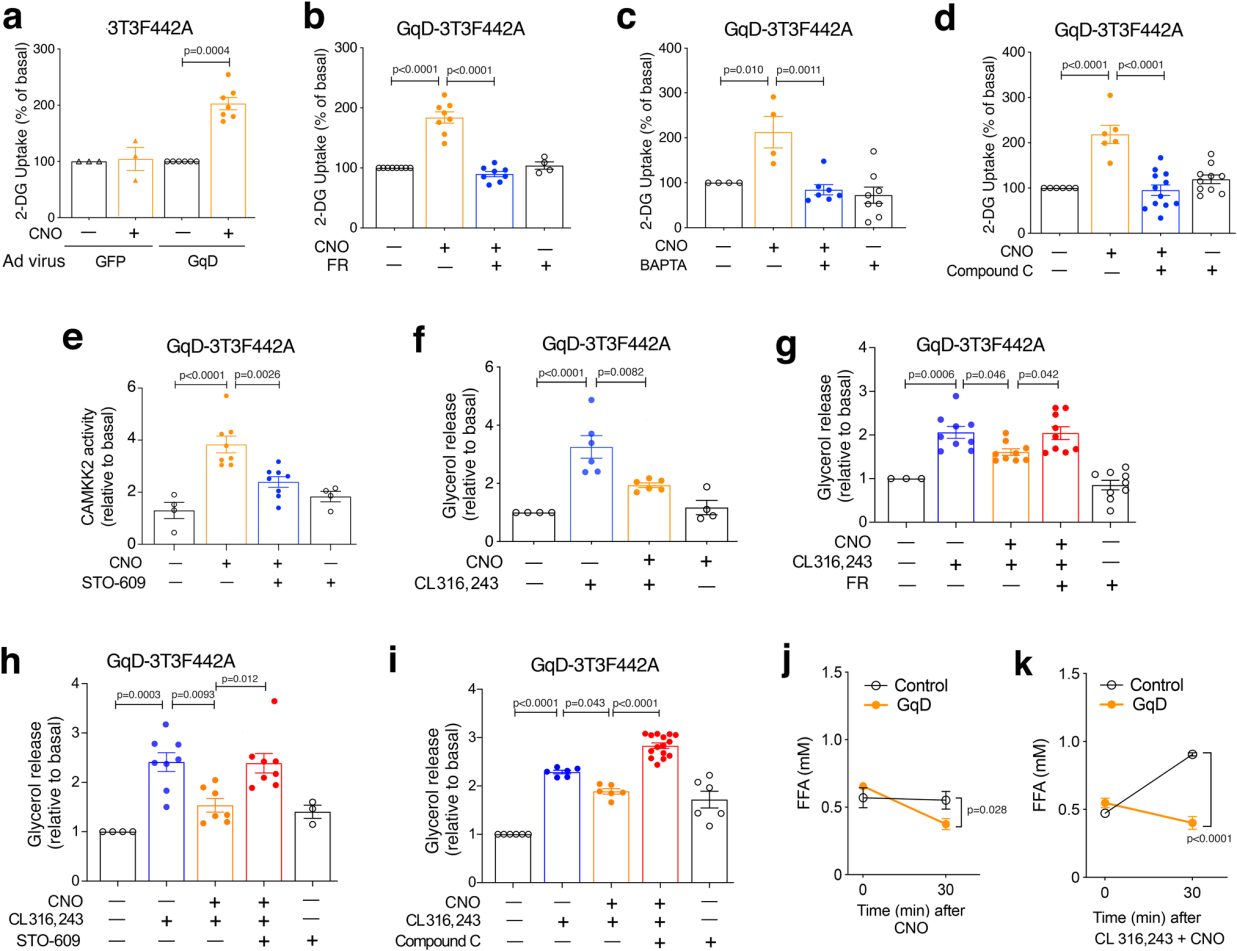
Activation of G_q_ signaling promotes glucose uptake and inhibits lipolysis in 3T3F442A adipocytes. **a** ^14^C-2-Deoxy-D-glucose (2-DG) uptake in GFP- or GqD-expressing 3T3F442A cells after treatment with CNO (10 μM). **b**–**d**, 2-DG uptake by CNO (10 μM)-treated GqD-expressing 3T3F442A cells in the presence or absence of the G_q/11_ inhibitor FR900359 (FR; 1 μM) (**b**), BAPTA (Ca^2+^ chelator, 10 μM) (**c**), or Compound C (AMPK inhibitor, 10 μM) (**d**). **e** CAMKK2 activity of GqD-expressing 3T3F442A cells after treatment with CNO (10 μM) and/or STO-609 (CAMKK2 inhibitor, 2 μM). **f** Glycerol release after treatment of GqD-expressing 3T3F442A cells with CL316,243 (100 nM) in the presence or absence of CNO (10 μM). **g**–**i** Glycerol release after treatment of GqD-expressing 3T3F442A cells with CL316,243 (100 nM), CNO (10 μM), and various pharmacological inhibitors (**g**: FR, 1 μM; **h**: STO-609, 2 μM; **i**: Compound C, 10 μM).). **j** Plasma FFA levels in adipo-GqD (GqD) and control mice before and after acute CNO injection (10 mg/kg i.p.) (*n* = 4-8 per group). **k** Plasma FFA levels in adipo-GqD (GqD) and control mice before and after i.p. treatment with CL316,243 (0.1 μg/kg) plus CNO (10 mg/kg) (*n* = 8 per group). In (**a**–**i**), data were normalized relative to values obtained in the absence of any drugs. Data are presented as means ± s.e.m. of at least three independent experiments (**a**–**i**). **a**, **j**, **k**: two-tailed Student’s *t*-test; **b**–**i**: one-way ANOVA followed by Bonferroni’s post-hoc test). Source data are provided as a Source data file.

We next examined whether CNO treatment was able to promote glucose uptake in differentiated GqD-3T3F442A cells, using the ^14^C-2-deoxy-D-glucose (2-DG) method. While CNO (10 μM) had no significant effect on glucose uptake in GFP-3T3F442A control cells, it caused a ~twofold increase in glucose uptake in GqD-3T3F442A cells (Fig. 4a). The stimulatory effect of GqD activation on glucose uptake was abolished by incubation of GqD-3T3F442A cells with FR900359 (G_q/11_ inhibitor; 1 μM), BAPTA (Ca^2+^ chelator, 10 μM), and Compound C (AMPK inhibitor, 10 μM) (Fig. 4b-d). We also noted that CNO treatment of GqD-3T3F442A cells stimulated the activity of CaM kinase (CaMK) kinase 2 (CAMKK2) by ~4-fold (Fig. 4e). This stimulatory CNO effect could be greatly reduced by incubation of GqD-3T3F442A cells with STO-609 (2 μM), a selective inhibitor of CAMKK2 (Fig. 4e). These observations support a model in which G_q_-induced glucose uptake by adipocytes depends on the known ability of activated G_q/11_ to increase intracellular Ca^2+^ levels^13^, triggering the activation of CAMKK2 and CAMKK2-mediated phosphorylation and activation of AMPK^32^.

### G_q_-mediated inhibition of lipolysis in mouse GqD-3T3F442A adipocytes

We carried out additional studies to explore the signaling pathway(s) mediating GqD-mediated inhibition of lipolysis. Treatment of GqD-3T3F442A adipocytes with CL316,243 (100 nM) caused a pronounced increased in lipolysis, as determined by the amount of glycerol secreted into the medium (Fig. 4f). The lipolytic effect of CL316,243 is known to be primarily due to G_s_-mediated activation of PKA^33^. The lipolytic response to CL316,243 was greatly reduced by co-incubation of GqD-3T3F442A cells with CNO (10 μM) (Fig. 4f). The ability of CNO to inhibit CL316,243-induced lipolysis was abolished by treatment of cells with FR900359 (1 μM) (Fig. 4g), confirming that CNO counteracts G_s_-stimulated lipolysis by activating G_q_-dependent signaling in GqD-3T3F442A cells. In addition, pharmacological blockade of CAMKK2 by STO-609 (2 μM) or inhibition of AMPK by Compound C (10 μM) abolished the ability of CNO-activated GqD receptors to reduce CL316,243-stimulated lipolysis in GqD-3T3F442A cells (Fig. 4h, i). CNO (10 μM) treatment of GFP-3T3F442A control cells had no significant effect on CL316,243-induced lipolysis (Supplementary Fig. 4d, e). These observations are in agreement with the data shown in Fig. 3, indicating that GqD-mediated activation of AMPK leads to the phosphorylation of HSL at a site that inhibits HSL activity.

Consistent with the in vitro data, CNO treatment (10 mg/kg i.p.) of adipo-GqD mice, but not of control littermates, caused a significant decrease of plasma FFA levels in vivo (Fig. 4j). Moreover, while CL316,243 (0.1 μg/kg i.p.) caused a ~2-fold increase in plasma FFA levels in control mice in vivo (Fig. 4k), this stimulatory CL316,243 response was absent in adipo-GqD mice treated with a mixture of CL316,243 and CNO (Fig. 4k). These findings further corroborate the concept that activation of adipocyte G_q_ signaling counteracts G_s_-stimulated lipolysis.

### Studies with differentiated human adipocytes

**Fig. 5:**
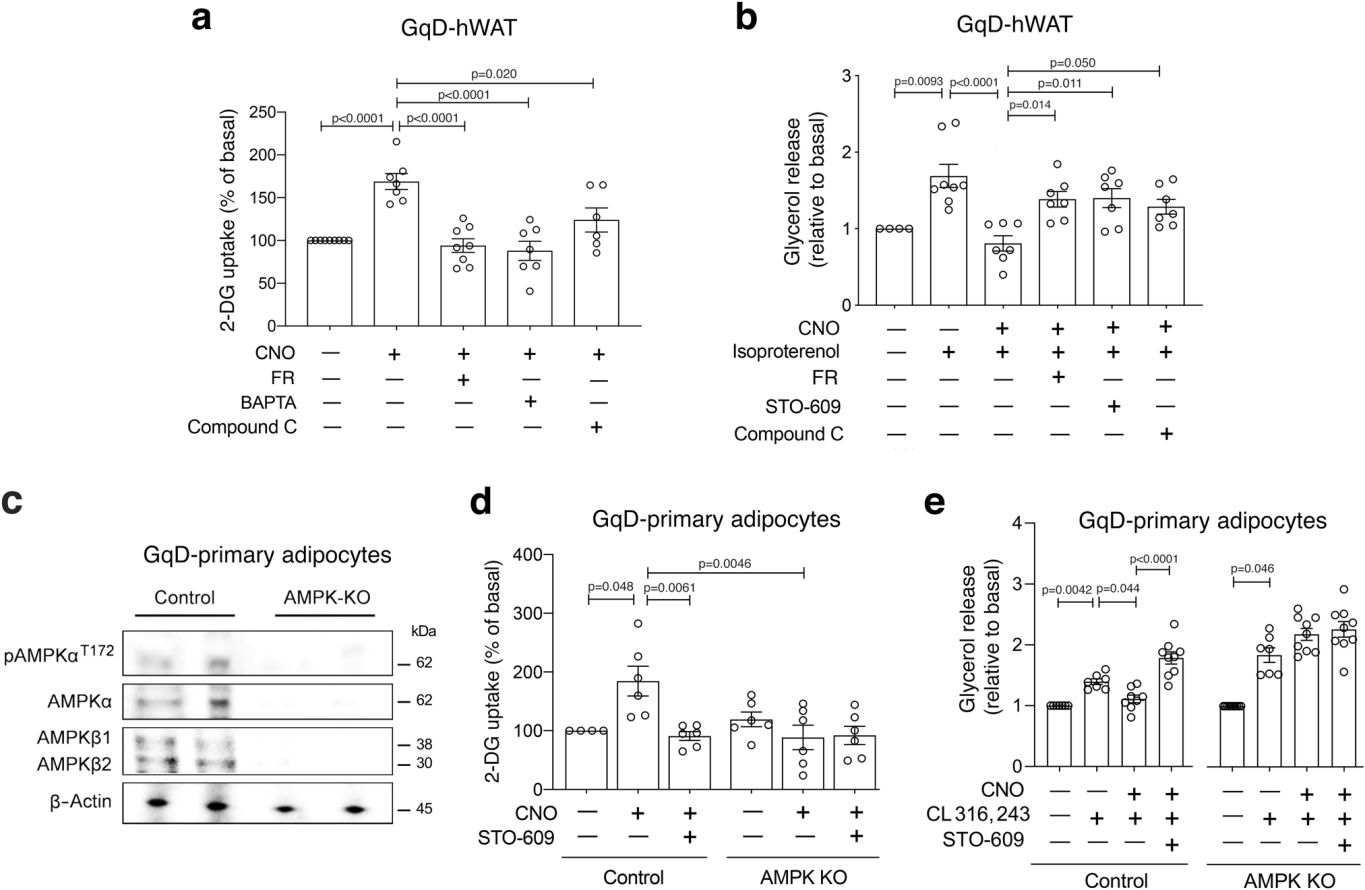
Studies with human adipocytes and primary mouse adipocytes lacking AMPK. **a** 2-Deoxy-D-glucose (2-DG) uptake by CNO (10 μM)-treated GqD-expressing, differentiated human white adipocytes (GqD-hWAT cells) in the presence or absence of FR900359 (FR; 1 μM), (BAPTA (10 μM), and Compound C (10 μM). **b** Glycerol release after treatment of GqD-hWAT cells with isoproterenol (100 nM), CNO (10 μM), and various pharmacological inhibitors (FR, 1 μM; STO-609, 2 μM; Compound C, 10 μM). **c** Western blot analysis with GqD-expressing primary mouse adipocytes (source: iWAT) demonstrating the lack of all three subunits of AMPK in AMPK KO cells (control = GqD-expressing primary mouse adipocytes with functional AMPK). This experiment was independently repeated twice with similar results. **d** 2-DG uptake by CNO (10 μM)-treated GqD-expressing primary mouse adipocytes in the presence (control) or absence of AMPK (AMPK KO), either with or without STO-609 (2 μM) co-incubation. **e** Glycerol release from CNO (10 μM)-treated GqD-expressing primary mouse adipocytes in the presence (control) or absence of AMPK (AMPK KO). Lipolysis was induced by incubation with CL316,243 (100 nM). Experiments were carried out in the absence or presence of STO-609 (2 μM). Glucose uptake and glycerol release data were normalized relative to values obtained in the absence of any drugs. Data are presented as means ± s.e.m. of at least four independent experiments. **a**, **b**, **d**, **e**: one-way ANOVA followed by Bonferroni’s post-hoc test). Source data are provided as a Source data file.

We next examined whether the metabolic effects observed after activation of adipocyte G_q_ signaling in mice are also conserved in human adipocytes. Specifically, we carried out glucose uptake and lipolysis assays with immortalized, differentiated human white adipocytes (hWAT cells)^34^ infected with an adenovirus coding for GqD (GqD-hWAT cells). As observed with mouse adipocytes, CNO (10 μM) treatment of GqD-hWAT cells caused a significant increase in glucose uptake (Fig. 5a). This stimulatory CNO effect was abolished by treatment of GqD-hWAT cells with FR900359 (1 μM), BAPTA (10 μM), or Compound C (10 μM) (Fig. 5a), indicating that activation of G_q_ signaling promotes glucose uptake via a similar mechanism in mouse and human adipocytes.

While the β3-AR is the predominant β-AR subtype in mouse adipocytes^35^, human adipocytes primarily express β1- and β2-ARs^36^. Treatment of differentiated GqD-hWAT cells with isoproterenol (100 nM), a non-subtype-selective β-AR agonist, led to a significant stimulation of lipolysis (Fig. 5b). This isoproterenol effect was absent in GqD-hWAT cells treated with a mixture of isoproterenol (100 nM) and CNO (10 μM) (Fig. 5b). CNO was unable to inhibit isoproterenol-stimulated lipolysis in GqD-hWAT cells in the presence of FR900359 (1 μM), STO-609 (2 μM), or Compound C (10 μM) (Fig. 5b). These data indicate that receptor-mediated activation of G_q_ signaling inhibits lipolysis in both mouse and human adipocytes via a CAMKK2/AMPK-dependent pathway.

### Studies with primary mouse adipocytes lacking AMPK

To demonstrate in a more direct fashion that AMPK is required for G_q_-mediated glucose uptake and inhibition of lipolysis, we carried out additional assays with primary mouse adipocytes (differentiated from pre-adipocytes) lacking AMPK. Specifically, we prepared adipocytes (pre-adipocytes) from iWAT of *adipoq-CreER*^*T2*^*-AMPK β1*^*flox/flox*^*β2*^*flox/flox*^ mice^37^ in which the two β subunits of AMPK were deleted by tamoxifen treatment^37^. Subsequently, the adipocytes were infected with an adenovirus encoding GqD. Since the β1 and β2 subunits are essential for AMPK heterotrimer formation, the absence of the two β subunits also resulted in a nearly complete loss of the expression of AMPKα, including its active form, p(T172)-AMPKα^37^ (Fig. 5c). For the sake of simplicity, we refer to these AMPK-deficient, GqD-expressing adipocytes simply as GqD-AMPK-KO adipocytes.

In agreement with the analysis of GqD-expressing 3T3-F442A adipocytes (Fig. 4a-d), CNO (10 μM) treatment of GqD-expressing primary adipocytes (differentiated from preadipocytes) prepared from control mice led to a ~2-fold increase in glucose uptake, an effect that could be completely blocked by pharmacological inhibition of CAMKK2 by STO-609 (2 μM) (Fig. 5d). In contrast, CNO was unable to stimulate glucose uptake in GqD-AMPK-KO adipocytes (Fig. 5d), providing additional strong evidence that G_q_-mediated activation of glucose uptake in adipocytes requires the presence of AMPK.

Lipolysis assays showed that CNO (10 μM) treatment of GqD-primary adipocytes inhibited the increase in lipolysis caused by CL316,243 (100 nM)-induced activation of β3-ARs (Fig. 5e). This CNO effect was completely absent after STO-609 (2 μM)-mediated inhibition of CAMKK2 (Fig. 5e). These findings mirror the data obtained with GqD-expressing 3T3-F442A adipocytes (Fig. 4f-i). Importantly, CNO treatment failed to inhibit CL316,243-induced lipolysis in GqD-AMPK-KO adipocytes (Fig. 5e). This observation further corroborates the concept that G_q_-mediated inhibition of lipolysis requires the activity of AMPK.

### Metabolic studies with mutant mice lacking Gα_q/11_ selectively in adipocytes

**Fig. 6:**
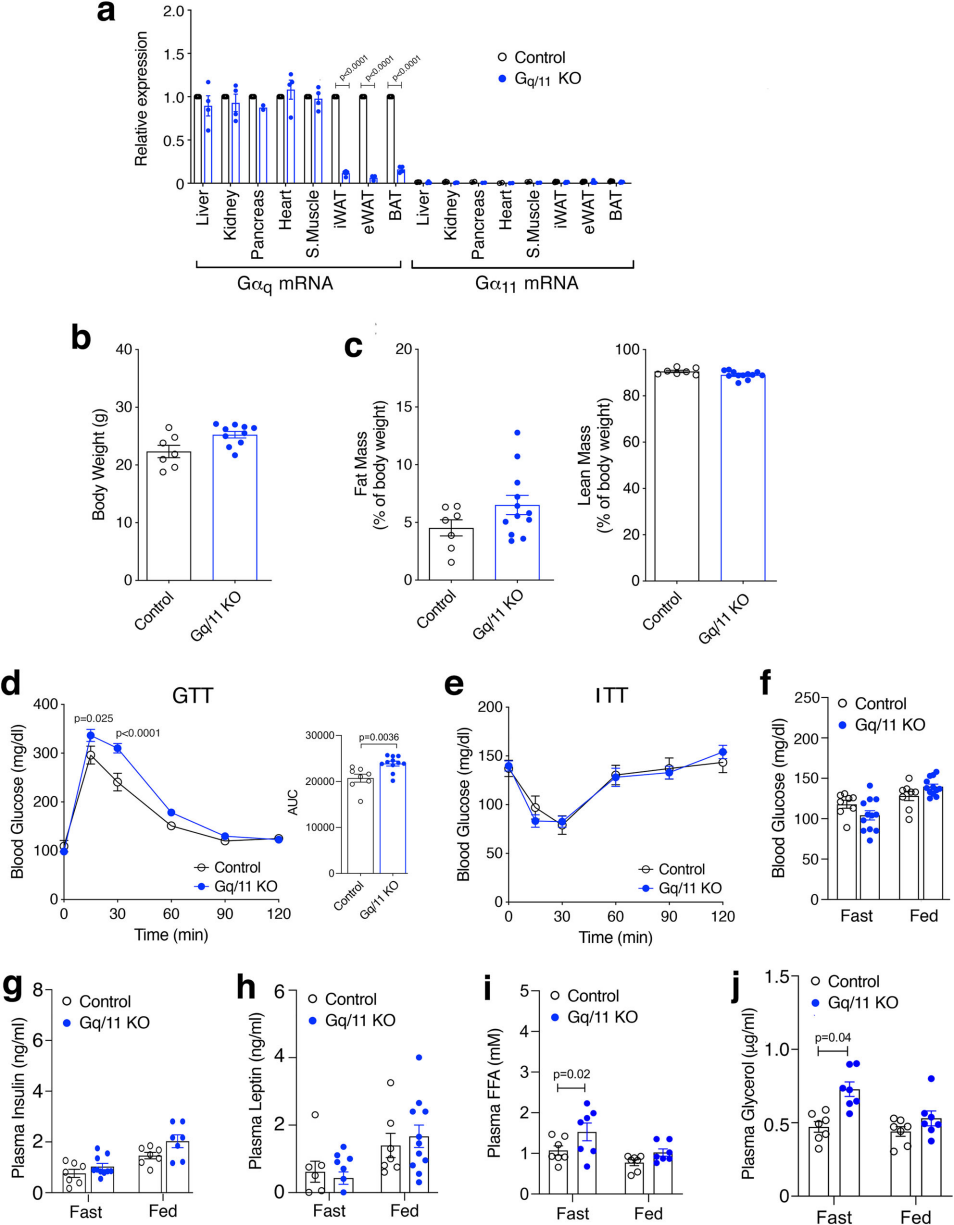
Adipocyte-selective G_q/11_ KO mice show deficits in blood glucose and lipid homeostasis. **a** Expression levels of *Gα*_*q*_ and *Gα*_*11*_ mRNA in different tissues of adipo-G_q/11_ KO mice and control littermates (*Gα*_*q*_*flox/flox Gα*_*11*_ − */−* mice lacking the *adipoq-Cre* transgene) (*n* = 4 per group). Transcript levels were normalized relative to G*α*_*q*_ mRNA levels of control mice (set equal to 1). **b**, **c** Body weight (**b**) and body composition (fat and lean mass as % of body weight) (**c**) of adipo-G_q/11_ KO mice and control littermates (mouse age: 12 weeks; n = 7–12 per group). **d**, **e** GTT (**d**; the panel to the right shows AUC values) and ITT (**e**) of adipo-G_q/11_ KO mice and control littermates. Blood glucose levels were measured after i.p. injection of 2 g/kg glucose after overnight fasting (**d**) or of 0.75 U/kg insulin after a 4 hr fast (**e**), respectively (*n* = 8–13 per group). **f**–**i**, Blood glucose (**f**), and plasma insulin (**g**), leptin (**h**), FFA (**i**), and glycerol (**j**) levels of adipo-G_q/11_ KO and control mice under fasting and fed conditions (*n* = 7–12 per group). Data are presented as means ± s.e.m. **d**, **e**: two-way ANOVA followed by Bonferroni’s post-hoc test; **a**, **f**–**j**: two-tailed Student’s t-test). Source data are provided as a Source data file.

The α-subunits of G_q_ and G_11_ (Gα_q_ and Gα_11_, respectively) are structurally closely related and have similar functional roles^13^. Gα_q_ and Gα_11_ are co-expressed in virtually all cell types including adipocytes^13^. Whole-body *G*α_*q*_ knockout (KO) mice display multiple peripheral and central deficits, while whole-body *G*α_*11*_ KO mice appear phenotypically normal^13^. To explore the role of G_q/11_ signaling in different cell types in vivo, Wettschureck et al. previously generated *Gα*_*q*_*flox/flox Gα*_*11*_ − */−* mice^38^. By crossing these mutant mice with *adipoq-Cre* mice, we obtained mutant mice lacking G_q/11_ in adipocytes (adipo-G_q/11_ KO mice) (Fig. 6a).

H&E staining of iWAT, eWAT, and BAT sections from adipo-G_q/11_ KO mice and control littermates did not reveal any obvious genotype-dependent morphological differences (Supplementary Fig. 7a). Moreover, the weights of different adipose tissue depots (expressed as % of body weight) did not differ significantly between the two groups of mice (Supplementary Fig. 7b).

Adipo-G_q/11_ KO mice did not differ from their control littermates (*Gα*_*q*_*flox/flox Gα*_*11*_ − */−* mice lacking the *adipoq-Cre* transgene) in body weight and composition (% fat and % lean mass), fed and fasting blood glucose and plasma insulin and leptin levels, as well as insulin sensitivity measured in an insulin tolerance test (Fig. 6b, c, e-h). However, adipo-G_q/11_ KO mice displayed significantly impaired glucose tolerance (Fig. 6d), as well as increased fasting plasma FFA and glycerol levels, as compared to their control littermates (Fig. 6i, j).

To induce obesity-associated metabolic deficits, we maintained adipo-G_q/11_ KO mice and their control littermates on a HFD for 8 weeks (Supplementary Fig. 8a). We found that HFD adipo-G_q/11_ KO mice showed significantly higher fasting blood glucose and plasma FFA levels than HFD control mice (Supplementary Fig. 8b, c). Moreover, glucose tolerance was severely impaired in HFD adipo-G_q/11_ KO mice, as compared to HFD control littermates (Supplementary Fig. 8f). Taken together, these observations clearly indicate that signaling via G_q/11_ proteins endogenously expressed by adipocytes plays a physiological role in maintaining proper glucose and lipid homeostasis.

### Insulin action is not affected by the lack of adipocyte G_q/11_ signaling

Previously published data involving the use of cultured 3T3-L1 cells suggested that G_q_-mediated signaling is involved in mediating insulin-induced effects^39^. To further explore this link, we injected adipo-G_q/11_ KO mice and control littermates (*Ga*_*q*_*flox/flox Ga*_*11*_ − */−* mice lacking the *adipoq-Cre* transgene) with insulin (0.75 U /kg i.p.), followed by the measurement of blood glucose and plasma FFA levels 30 min later. We found that insulin treatment reduced blood glucose and plasma FFA levels to a similar degree in control and adipo-G_q/11_ KO mice (Supplementary Fig. 9a, b). Moreover, studies with 3T3F442A cells showed that the ability of insulin (10 nM) to stimulate glucose uptake and inhibit β3-adrenergic receptor (CL316,243)-mediated lipolysis was not significantly different in the presence or absence of FR900359 (FR; G_q/11_ inhibitor; 1 μM) (Supplementary Fig. 9c, d). Taken together, our data suggest that insulin action on adipocytes does not require the presence of adipocyte G_q/11_ under our experimental conditions.

### G_q_-coupled receptors endogenously expressed by adipocytes

Since activation or disruption of G_q_ signaling in adipocytes had significant effects on glucose uptake and lipolysis, we speculated that targeting G_q_-coupled receptors endogenously expressed by adipocytes might prove beneficial to treat impairments in glucose and lipid homeostasis. We recently subjected RNA prepared from isolated mouse adipocytes (iWAT and eWAT) to RNA-seq analysis^14^. This analysis demonstrated that mouse adipocytes express several GPCRs that are selectively coupled to G_q/11_, including the P2Y_6_ receptor, the complement component 5a receptor 1, the cysteinyl leukotriene receptors 1 and 2, and several orphan GPCRs (Supplementary Table 1). Interestingly, the expression levels of several of these receptors were upregulated when mice were maintained on a HFD (Supplementary Table 1).

Amisten et al.^11^ recently published a comprehensive list of GPCRs expressed by human subcutaneous fat (abdominal and gluteofemoral fat depots). The most abundantly expressed G_q_-coupled receptors for which quantitative expression data were provided are listed in Supplementary Table 2. A comparison of the receptors listed in Supplementary Tables 1 and 2 indicates that the cysteinyl leukotriene receptor 2 (CysLT_2_ receptor) is expressed in both mouse adipocytes and in human subcutaneous fat.

### CysLT_2_ receptor function in human adipocytes

**Fig. 7:**
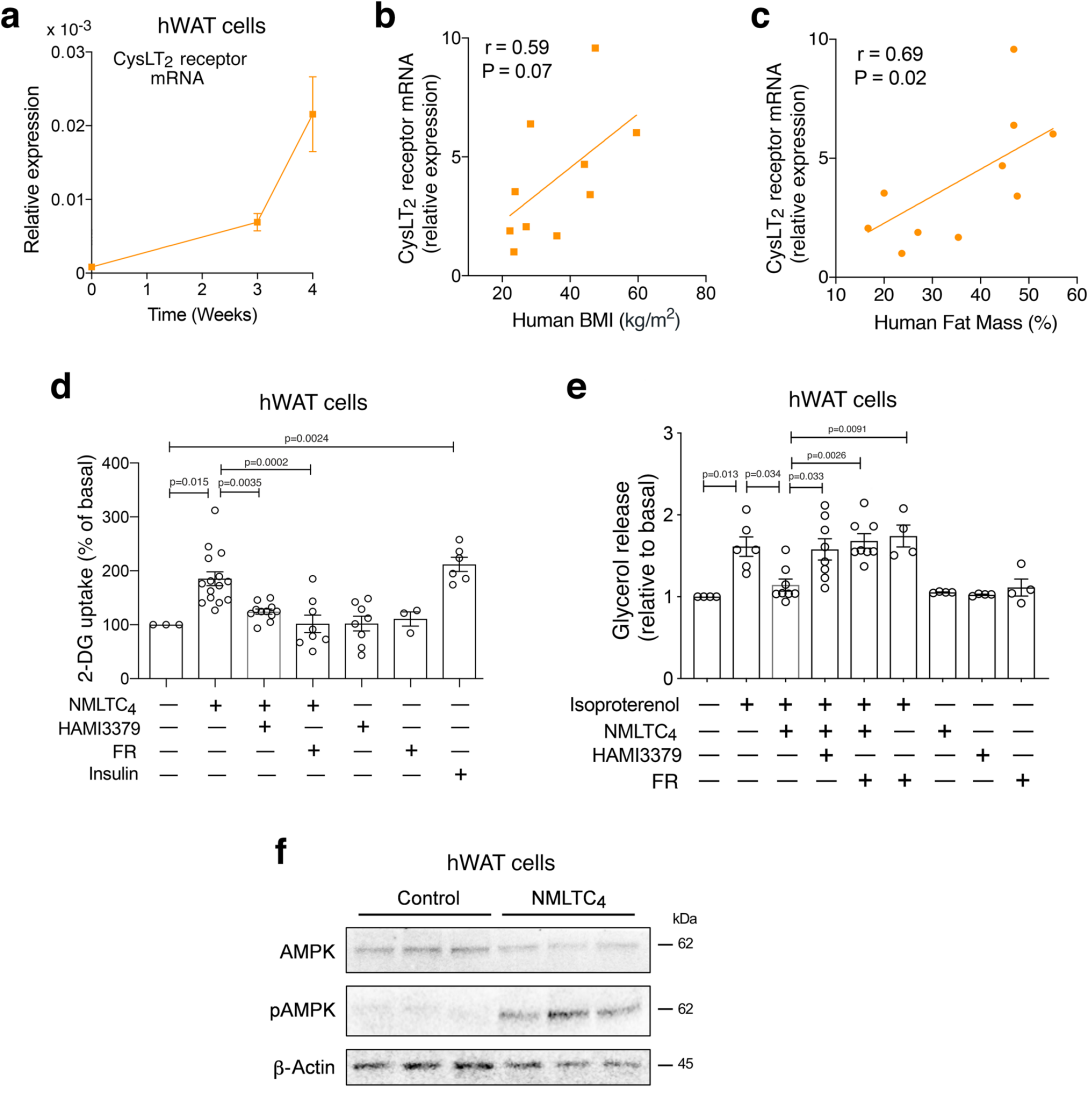
Agonist stimulation of the CysLT_2_ receptor promotes glucose uptake and inhibits lipolysis in human white adipocytes. **a** Changes in mRNA levels of the CysLT_2_ receptor during the differentiation of human white adipocytes (hWAT cells). **b** Correlation between human body mass index (BMI) and CysLT_2_ receptor mRNA expression levels in human subcutaneous fat (*n* = 10; samples were from 10 different individuals). **c** Correlation between human fat mass (% of body weight) and CysLT_2_ receptor mRNA expression levels in human subcutaneous fat (*n* = 10; samples were from 10 different individuals). **d** Stimulation of 2-deoxy-D-glucose (2-DG) uptake by differentiated hWAT cells by NMLTC_4_, a selective CysLT_2_ receptor agonist (1 μM). HAMI3379, a selective CysLT_2_ receptor antagonist (1 μM) and FR900359 (FR; G_q/11_ inhibitor; 1 μM) blocked this response. For control purposes, cells were also treated with insulin (10 nM). **e** Stimulation of glycerol release from differentiated hWAT cells by isoproterenol (100 nM). This effect was completely blocked by NMLTC_4_ (1 μM). The anti-lipolytic effect of NMLTC_4_ was absent in the presence of HAMI3379 (1 μM) or FR (1 μM). Glucose uptake and glycerol release data were normalized relative to values obtained in the absence of any drugs. **f** Western blotting studies with differentiated hWAT cells. Treatment with NMLTC_4_ (1 μM) for 60 min promotes the phosphorylation of AMPK (α-subunit). This experiment was independently repeated twice with similar results. The correlation analysis shown in (**b**, **c**) was conducted by Spearman’s test (two-tailed, no adjustment for multiple comparisons). The data shown in (**a**, **d**, **e**) are means ± s.e.m. obtained in at least three independent experiments. In (**d**, **e**), data were expressed relative to basal levels measured in the absence of any drugs (one-way ANOVA followed by Bonferroni’s post-hoc test). Source data are provided as a Source data file.

We next studied the potential functional roles of the CysLT_2_ receptor in human adipocytes. Fig. 7a shows that CysLT_2_ receptor expression (mRNA levels) dramatically increases during the differentiation of human white adipocytes (hWAT cells). Moreover, studies with lean human subjects and individuals with obesity demonstrated that CysLT_2_ receptor transcript levels in subcutaneous fat from biopsy samples^40^ correlated well with body mass index (BMI) and fat mass (% of body weight) (Fig. 7b, c), suggesting that adipocyte CysLT_2_ receptors may play a role in regulating the function of human WAT.

To further explore this concept, we treated differentiated hWAT cells with N-methyl leukotriene C_4_ (NMLTC_4_), a selective CysLT_2_ receptor agonist^41^. NMLTC_4_ is a metabolically more stable analog of leukotriene C_4_ (LTC_4_) and activates both mouse and human CysLT_2_ receptors with high selectivity in vitro and in vivo^41^. Incubation of hWAT cells with NMLTC_4_ (1 μM) led to a ~twofold increase in glucose uptake (Fig. 7d). The magnitude of this effect was similar to that observed after treatment of hWAT cells with insulin (10 nM) (Fig. 7d). The NMLTC_4_-stimulated increase in glucose uptake could be blocked by HAMI3379 (1 μM), a potent and selective blocker of CysLT_2_ receptors^42^, as well as FR900359 (1 μM), indicating that activation of human adipocyte CysLT_2_ receptors promotes glucose uptake via stimulation of G_q/11_ signaling (Fig. 7d).

Studies with differentiated hWAT cells also showed that NMLTC_4_ (1 μM) effectively inhibited the increase in lipolysis caused by isoproterenol (100 nM), a nonsubtype-selective β-AR agonist (Fig. 7e). This inhibitory NMLTC_4_ effect was not observed in the presence of the CysLT_2_ receptor antagonist, HAMI3379 (1 μM), or the G_q/11_ inhibitor, FR900359 (1 μM) (Fig. 7e). These findings clearly indicate that adipocyte CysLT_2_ receptors mediate inhibition of lipolysis in human adipocytes via activation of G_q/11_ signaling.

In agreement with the data obtained with CNO-treated, GqD-expressing mouse adipocytes (Fig. 3a, b), NMLTC_4_ treatment of hWAT cells also efficiently promoted the phosphorylation of AMPK (Fig. 7f).

### Activation of adipocyte G_q_ signaling overcomes metabolic deficits caused by adipocyte insulin receptor deficiency

It is known that insulin resistance of adipose tissue plays a key role in the pathogenesis of T2D^43^. In agreement with this concept, mutant mice lacking insulin receptors (IRs) selectively in adipocytes develop profound insulin resistance and hyperglycemia^44^. Our in vitro data obtained with cultured mouse and human adipocytes indicated that receptor-mediated activation of G_q/11_ was able to stimulate glucose uptake and inhibit lipolysis by adipocytes in an insulin-independent fashion. We therefore hypothesized that activation of adipocyte G_q/11_ signaling might compensate for impaired insulin signaling in adipocytes. To test this hypothesis, we generated adipo-GqD mice that carried only one copy of the IR gene in adipocytes (adipo-GqD-IR^+/−^ mice; heterozygous, adipocyte-specific IR KO mice expressing GqD in adipocytes). As shown in Supplementary Fig. 10, the expression of IR mRNA was reduced by ~50% in adipose tissues isolated from adipo-GqD-IR^+/−^ mice, as compared to their control littermates (LSL-GqD-IR^+/flox^ mice that do not carry the *adipoq-Cre* transgene).

**Fig. 8:**
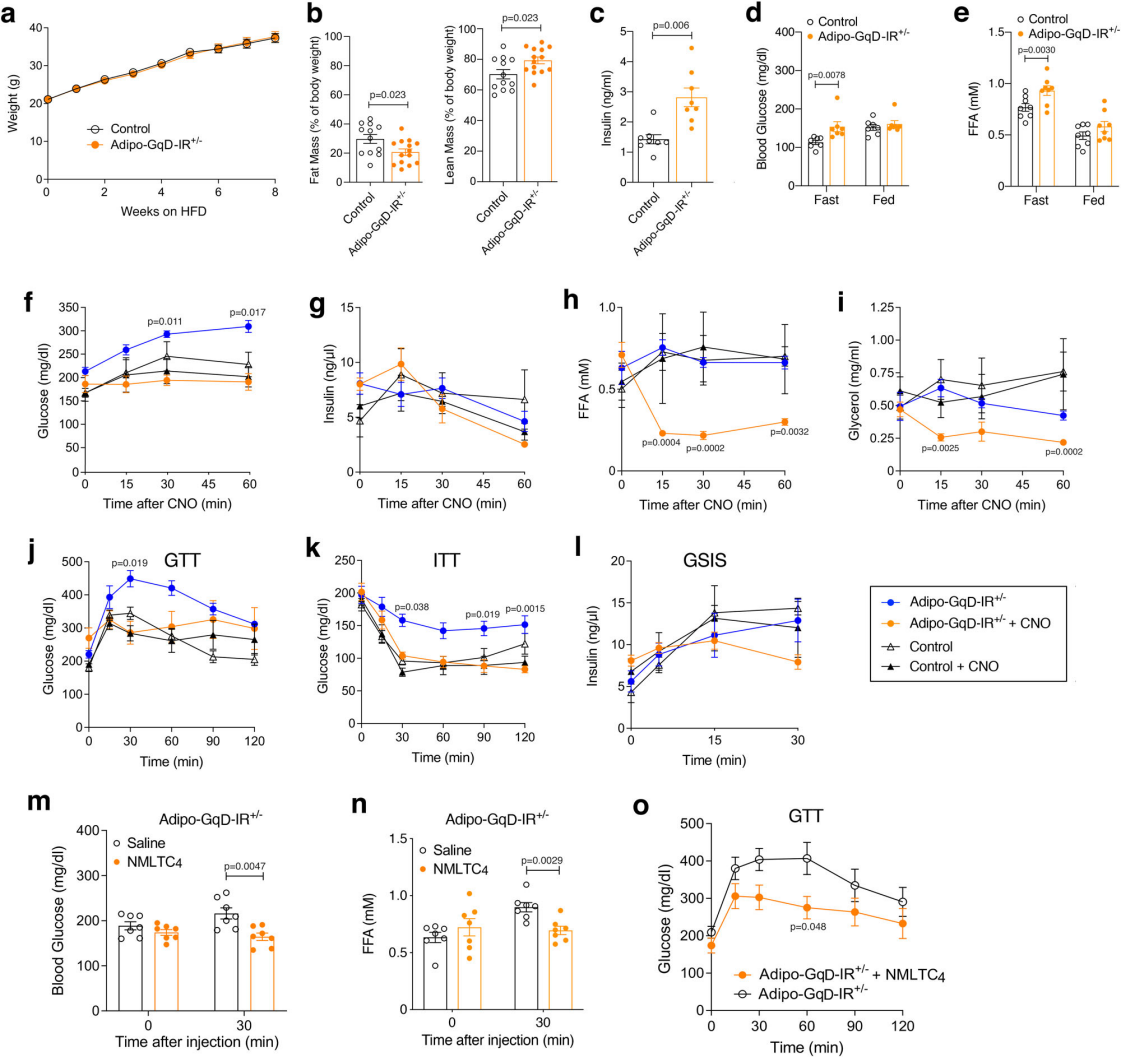
Activation of adipocyte G_q_ signaling restores normal glucose and lipid homeostasis in adipocyte-specific insulin receptor (IR) mutant mice. **a** Body weight gain of adipo-GqD-IR^+/-^ mice (adipocyte-specific heterozygous insulin receptor knockout mice expressing GqD in adipocytes) and control littermates (LSL-GqD-IR^+/flox^ mice that do not carry the *adipoq-Cre* transgene) maintained on a high fat diet (HFD). **b**–**e** Body composition (fat and. lean body mass) (**b**), plasma insulin levels after a 16 hr overnight fast (**c**), and fed and fasting (16 hr overnight) blood glucose (**d**) and plasma FFA levels (**e**) of adipo-GqD-IR^+/-^ mice and control littermates maintained on HFD for ~10 weeks. **f**–**i**, Blood glucose (**f**), plasma insulin (**g**), plasma FFA (**h**), and plasma glycerol (**i**) levels of HFD adipo-GqD-IR^+/-^ and control mice after acute treatment with CNO (10 mg/kg i.p.) following a 4 hr fast. **j**–**l**, GTT (**j**), ITT (**k**), and GSIS (**l**) performed with HFD adipo-Gq-IR^+/-^ mice and control littermates with or without CNO (10 mg/kg i.p.) treatment. In (**j**), mice were injected with 1 g/kg glucose (i.p.) after a 4 h fast. In (**k**) and (**l**), mice that had been fasted for 4 hr received 1 U/kg insulin (i.p) or a bolus of glucose (1 g/kg i.p.), respectively. **m**–**o**, NMLTC_4_ treatment of HFD adipo-GqD-IR^+/-^ mice. **m**, **n**, Blood glucose (**m**) and plasma FFA (**n**) levels of HFD adipo-GqD-IR^+/-^ mice after treatment with NMLTC_4_ (10 μg/kg) or saline following a 4 h fast. **o**, GTT. After a 4 h fast, HFD adipo-GqD-IR^+/-^ mice were injected with glucose (1 g/kg i.p.), either in the presence or absence of NMLTC_4_ (10 μg/kg). All experiments were carried out with male mice maintained on HFD for 8–14 weeks Data are presented means ± s.e.m. (**a**, **b**: *n* = 10-14 per group; **c**–**o**: n = 5-8 per group). **b**–**e**, **m**, **n**: two-tailed Student’s *t*-test; **f**–**l**, **o**: two-way ANOVA followed by Bonferroni’s post hoc test) (**f**–**l**: adipo-GqD-IR^+/-^ plus CNO vs. adipo-GqD-IR^+/-^ without CNO). Source data are provided as a Source data file.

When maintained on a HFD, adipo-GqD-IR^+/−^ mice showed similar body weight gain as their control littermates (Fig. 8a). However, after 8 weeks of HFD feeding, adipo-GqD-IR^+/−^ mice displayed a significant reduction in body fat mass, accompanied by a significant increase in lean mass (Fig. 8b). Although plasma insulin levels were increased in fasted adipo-GqD-IR^+/−^ mice (Fig. 8c), fasting blood glucose and plasma FFA levels were significantly elevated in adipo-GqD-IR^+/−^ mice (Fig. 8d, e), indicating that reduced IR signaling in adipocytes causes impaired glucose and lipid homeostasis. To investigate whether activation of adipocyte G_q_ signaling was able to ameliorate these metabolic deficits, we injected HFD adipo-GqD-IR^+/−^ mice and their control littermates with a single i.p. dose of CNO (10 mg/kg) or vehicle (saline). Vehicle-treated adipo-GqD-IR^+/−^ mice showed a pronounced increase in blood glucose levels, most likely due to stress-induced glucose mobilization (Fig. 8f). This effect was not observed when adipo-GqD-IR^+/−^ mice received CNO instead of vehicle (Fig. 8f). Plasma insulin levels remained largely unchanged in adipo-GqD-IR^+/−^ and control mice after vehicle or CNO administration (Fig. 8g). Strikingly, CNO treatment of adipo-GqD-IR^+/−^ mice, but not of control littermates, led to a pronounced reduction in plasma FFA and glycerol levels (Fig. 8h, i), indicating that activation of adipocyte G_q/11_ signaling inhibits lipolysis in this mouse model of insulin resistance. As expected, this CNO effect was not observed with control mice that did not express the GqD designer receptor (Fig. 8h, i).

HFD adipo-GqD-IR^+/−^ mice also showed significant impairments in glucose tolerance and peripheral insulin sensitivity, as compared with their HFD control littermates (Fig. 8j, k). Notably, co-administration of glucose (1 g/kg i.p.) or insulin (1 U/kg i.p.) with CNO (10 mg/kg i.p.) greatly improved glucose tolerance and insulin sensitivity caused by the relative lack of IRs in adipocytes (Fig. 8j, k). Expectedly, CNO administration had no significant effect on glucose tolerance and insulin sensitivity in HFD control littermates (Fig. 8j, k). Administration of a glucose bolus (1 g/kg i.p.) triggered comparable increases in plasma insulin levels in adipo-GqD-IR^+/−^ and control mice, either in the presence or absence of co-injected after CNO (Fig. 8l).

### Treatment with a CysLT_2_ receptor agonist ameliorates the metabolic deficits displayed by adipo-GqD-IR^+/^ mice

Since CysLT_2_ receptors are expressed in both mouse and human adipocytes/fat tissues (Supplementary Tables 1 and 2) and NMLTC_4_, a selective CysLT_2_ receptor agonist, promoted glucose uptake and inhibited lipolysis in cultured hWAT cells (Fig. 7d, e), we next injected HFD adipo-GqD-IR^+/−^ mice with a single i.p. dose of NMLTC_4_ (10 μg/kg i.p.) or vehicle (saline). Thirty minutes later, the NMLTC_4_-treated adipo-GqD-IR^+/−^ mice showed significantly decreased blood glucose and plasma FFA levels, as compared to the vehicle-treated adipo-GqD-IR^+/−^ mice (Fig. 8m, n). Moreover, a GTT showed that co-administration of glucose (1 g/kg i.p.) with NMLTC_4_ (10 μg/kg i.p.) greatly improved the deficit in glucose tolerance displayed by HFD adipo-GqD-IR^+/−^ mice (Fig. 8o).

### The beneficial metabolic effects caused by CysLT_2_ receptor activation require adipocyte G_q/11_ signaling

To explore whether the beneficial metabolic effects caused by NMLTC_4_ treatment were mediated by adipocyte G_q/11_ signaling, we next injected HFD adipo-G_q/11_ KO and control mice with either vehicle (saline) or NMLTC_4_ (10 μg/kg i.p.). Injection with saline caused modest increases in blood glucose and plasma FFA levels in most mice (Supplementary Fig. 8d, e), most likely due to the injection stress. Injection of HFD adipo-G_q/11_ KO mice with NMLTC_4_ (10 μg/kg i.p.) resulted in similar responses (Supplementary Fig. 8d, e). In contrast, NMLTC_4_ treatment of HFD control mice did not cause increases in blood glucose and plasma FFA levels in most animals, suggesting that activation of adipocyte CysLT2 receptors counteracts the increases in blood glucose and plasma FFA levels caused by the injection stress.

Importantly, while NMLTC_4_ treatment significantly improved glucose tolerance in HFD control mice, NMLTC_4_ had no effect on the severe impairment in glucose tolerance displayed by HFD adipo-G_q/11_ KO mice (Supplementary Fig. 8f, g). Taken together, these data indicate that the beneficial metabolic effects of NMLTC_4_ treatment were mediated by adipocyte CysLT2 receptors linked to the activation of G_q/11_.

### G_q/11_ signaling is elevated in adipose tissue-derived from fasted mice

To explore whether fasting affected the degree of G_q/11_ signaling in adipose tissues, we measured inositol monophosphate (IP_1_) levels in adipose tissues isolated from freely fed and fasted (overnight for 14 hr) male WT C57BL/6 mice (age: 8 weeks). Mice consuming regular chow were injected with LiCl (10 mmoles/kg, s.c.), an inhibitor of IP_1_ phosphatase, prior to the beginning of the dark cycle (6 pm). In the presence of LiCl, the intracellular accumulation of IP_1_ is considered a good measure of the degree of G_q/11_ signaling^45^.

We found that fasted mice showed ~2.7-fold higher IP_1_ levels in visceral fat (eWAT), as compared to mice that had free access to food (Supplementary Fig. 11). IP_1_ levels also trended to be increased in subcutaneous fat (iWAT), but this effect failed to reach statistical significance (Supplementary Fig. 11). One possible explanation for this latter observation is that G_q/11_-coupled receptors are expressed at lower levels in iWAT than in eWAT. In any case, the eWAT data support the concept that G_q/11_ signaling is elevated in adipocytes under fasting conditions.

## Discussion

Adipocyte function is regulated by many hormones, neurotransmitters, and signaling proteins, including GPCRs. Adipocytes express dozens of GPCRs that are linked to different functional classes of G proteins^11,12^. Previous in vivo studies have shown that selective activation of adipocyte G_s_ signaling leads to striking improvements in glucose homeostasis and protects mice from the metabolic deficits associated with the consumption of an obesogenic diet (see, for example, ref. ^15,16^). Mechanistic data indicated that these beneficial metabolic effects are due to the ability of adipocyte G_s_ signaling to stimulate lipolysis and energy expenditure and to inhibit food intake^15,16^. Moreover, a recent study demonstrated that adipocyte G_i_ signaling is essential for maintaining euglycemia and proper peripheral insulin sensitivity, primarily due to G_i_-mediated suppression of lipolysis and the resulting reduction of plasma FFA levels^14^.

In the present study, we provide strong evidence that drug-induced activation of adipocyte G_q_ signaling improves glucose homeostasis and peripheral insulin sensitivity under both physiological and pathophysiological conditions. By using a chemogenetic approach, we demonstrated that these beneficial metabolic effects could be observed after both acute and chronic activation of adipocyte G_q_ signaling (Figs. 1, 2; Supplementary Fig. 2).

In agreement with these findings, mice that lacked G_q/11_ in adipose tissue (adipo-G_q/11_ KO mice) showed significant impairments in glucose homeostasis, associated with elevated fasting plasma FFA levels (Fig. 6). Similar deficits were observed when adipo-G_q/11_ KO mice were maintained on an obesogenic diet (Supplementary Fig. 8). These data clearly indicate that endogenous adipocyte G_q/11_ signaling is required for maintaining euglycemia and normal plasma FFA levels. The precise physiological and pathophysiological roles of adipocyte G_q/11_ signaling remain to be explored in future studies.

While CNO treatment of adipo-GqD mice resulted in a striking decrease in blood glucose levels (Fig. 2c), fed and fasting blood glucose levels were similar in adipo-G_q/11_ KO and control mice consuming regular chow (Fig. 6f). This latter observation suggests that other signaling pathways can compensate for the lack of adipocyte G_q/11_ to maintain euglycemia. However, when challenged with an obesogenic diet, adipo-G_q/11_ KO showed significantly elevated fasting blood glucose levels (Supplementary Fig. 8b).

We also found that the G_q_-coupled CysLT_2_ receptor is enriched in mouse and human adipocytes (Fig. 7a, Supplementary Tables 1 and 2). In agreement with the data obtained with adipo-GqD mice, NMLTC_4_, a selective CysLT_2_ receptor agonist, inhibited lipolysis and increased glucose uptake in human adipocytes (Fig. 7d, e) and improved glucose and lipid homeostasis in a mutant mouse strain characterized by impaired insulin signaling in adipocytes (see next paragraph) (Fig. 8m-o).

NMLTC_4_ treatment of HFD adipo-G_q/11_ KO failed to improve glucose homeostasis (Supplementary Fig. 8f), strongly suggesting that the beneficial metabolic effects of NMLTC_4_ administration were mediated by adipocyte CysLT2 receptors linked to the activation of G_q/11_. Thus, this proof-of-concept study indicates that agonist activation of a G_q_-coupled receptor endogenously expressed by mouse and human adipocytes can lead to striking improvements in glucose homeostasis and other beneficial metabolic effects. In this context, it should be noted that gain of function mutations in the CysLT2 receptor are associated with various cancers (see, for example, ref. ^46^). For this reason, it is unlikely that CysLT2 agonists can be developed for clinical use.

Studies with cultured mouse and human adipocytes indicated that agents able to activate adipocyte G_q_ signaling mimic the major actions of insulin on adipocyte function, inhibition of lipolysis, and stimulation of glucose uptake. In agreement with these observations, several previous studies reported that activation of G_q_ signaling can promote glucose uptake and/or GLUT4 translocation^39,47,48^ and may inhibit lipolysis^49,50^ in cultured adipocytes. On the basis of these findings, we speculated that drugs able to activate G_q_ signaling in adipocytes in vivo may prove useful to overcome impaired glucose and lipid homeostasis caused by adipose tissue insulin resistance. To test this hypothesis, we generated insulin receptor (IR) mutant mice lacking one copy of the IR gene in adipocytes (adipo-IR^+/−^ mice). When maintained on an obesogenic diet (HFD), these mutant mice showed significantly increased fasting blood glucose and plasma FFA levels (despite increased plasma insulin levels), indicative of an insulin-resistant state (Fig. 8c-e). In agreement with these data, a recent study demonstrated that mutant mice lacking both copies of the IR gene selectively in adipocytes (adipo-IR^−/−^ mice) develop profound insulin resistance and hyperglycemia^44^.

Remarkably, chemogenetic activation of G_q_ signaling in adipocytes of HFD adipo-GqD-IR^+/−^ mice greatly improved the deficits in glucose tolerance and insulin sensitivity displayed by the IR mutant mice (Fig. 8h-k). These data strongly support the concept that drugs that can stimulate G_q_ signaling in adipocytes, such as agonists acting on G_q_-coupled receptors expressed by adipocytes, can overcome the metabolic deficits caused by impaired insulin action on adipose tissue. For this reason, our findings point to a new avenue to develop novel classes of antidiabetic agents.

To study the molecular mechanism underlying the beneficial metabolic effects of activating G_q_ signaling in adipocytes, we carried out studies with cultured mouse and human adipocytes. CNO treatment of mouse and human adipocytes (hWAT cells) expressing the GqD designer receptor impaired lipolysis and improved glucose uptake (Figs. 4 and 5). Both effects were either completely abolished or greatly reduced after treatment with FR900359 (selective G_q/11_ inhibitor), BAPTA (Ca^2+^ chelator), STO-609 (selective CAMKK2 inhibitor), and Compound C (selective AMPK inhibitor) (Figs. 4 and 5). Moreover, CNO was unable to promote glucose uptake and inhibit lipolysis in mouse primary adipocytes lacking AMPK (Fig. 5c-e), indicating that G_q_-induced activation of AMPK plays a central role in mediating these effects. The ability of activated G_q_ to stimulate AMPK has also been observed in other tissues and cell types^51–53^. Following agonist binding, G_q_-coupled receptors trigger increases in intracellular Ca^2+^ levels^10,13^, leading to the activation of CAMKK2 which then phosphorylates and activates AMPK^32^. Activated AMPK is able to inhibit lipolysis via phosphorylation of HSL at S565, an event that interferes with proper HSL function (Fig. 3g, h)^30^, and to promote glucose uptake by activating a signaling pathway that involves the phosphorylation of the Rab GTPase-activating protein AS160 (alternative name: Tbc1d4) that ultimately leads to the translocation of GLUT4 to the plasma membrane (Fig. 3a, b, e, f)^54^ (see Supplementary Fig. 12 for a summary of this signaling network).

Interestingly, a recent study showed that CNO treatment of murine brown preadipocytes expressing the GqD designer receptor suppressed lipogenesis^55^. In agreement with this finding, ligand-mediated activation of endogenously expressed, G_q_-coupled endothelin ET_1A_ receptors also inhibited the differentiation of brown adipocytes^55^. Mechanistic studies showed that G_q_ activation inhibits the differentiation of brown adipocytes via stimulation of the RhoA/ROCK pathway^55^. These observations underline the key role of G_q_ signaling in regulating the activity/differentiation of both white and brown adipocytes.

In a previous study, Imamura et al.^39^ reported that single-cell microinjection of a G*α*_q/11_ antibody into 3T3-L1 adipocytes markedly inhibited insulin-induced GLUT4 translocation, suggesting that efficient insulin-stimulated GLUT4 translocation in adipocytes requires G_q_ signaling. However, our data strongly suggest that agents that can activate G_q_ signaling can promote glucose uptake into adipocytes in an insulin-independent fashion. It should also be noted that Imamura et al.^39^ only studied cultured cells and did not explore the effect of G_q_ signaling on lipolysis.

By using adipo-G_q/11_ KO mice and FR900359 (FR; G_q/11_ inhibitor; 1 μM) as experimental tools, we demonstrated that the ability of insulin to stimulate glucose uptake by adipocytes and inhibit lipolysis did not require the presence of adipocyte G_q/11_ under our experimental conditions (Supplementary Fig. 9). However, this finding does not exclude the possibility that insulin signaling is regulated by the activity of G_q/11_ (or vice versa) in other cell types or physiological responses. The seemingly discrepant findings between the study by Imamura et al.^39^ and our data may be due to differences in experimental conditions including the use of different cell lines and alternative strategies to interfere with adipocyte G_q/11_ signaling.

IP_1_ assays indicated that G_q/11_ signaling was enhanced in adipose tissue (eWAT) of fasted WT mice (Supplementary Fig. 11). Since adipo-Gq/11 KO mice showed elevated plasma FFA levels under fasting conditions (Fig. 6i, Supplementary Fig. 8c), the IP_1_ data suggest that adipocyte G_q/11_ signaling plays a role in regulating the magnitude of fasting-induced lipolysis. As discussed above, activation of adipocyte G_q/11_ signaling led to improved glucose tolerance in several mouse models. Since all glucose tolerance tests were carried out with fasted mice, it is possible that enhanced adipocyte G_q/11_ signaling contributes to the removal of glucose from the blood, consistent with the notion that this pathway plays a role in maintaining glucose homeostasis.

In summary, by using a combined molecular genetic/biochemical/pharmacologic approach we provide strong evidence that activation of adipocyte G_q_ signaling in vitro and in vivo promotes glucose uptake and exerts a strong antilipolytic effect in an AMPK-dependent fashion. In a proof-of-concept study, we also demonstrate that agonist simulation of a G_q_-coupled receptor (CysLT_2_ receptor) endogenously expressed by adipocytes can overcome the metabolic deficits caused by impaired insulin receptor signaling in adipocytes. Importantly, our data clearly indicate that endogenous adipocyte G_q/11_ signaling plays a physiological role in maintaining proper glucose and lipid homeostasis. These findings should pave the way toward the development of GPCR-based therapies aimed at restoring impaired adipocyte function in T2D and related metabolic disorders.

## Methods

### Generation and maintenance of mutant mice

Mutant mice expressing a G_q_-coupled DREADD (hM3Dq; short name: GqD)^18^ selectively in adipocytes (adipo-GqD mice) were generated by crossing *adipoq-Cre* mice (The Jackson Laboratory, stock # 010803) with *LSL-hM3Dq* mice (alternative name: *CAG-LSL-hM3Dq* mice)^20^. Mice that carried one copy of the *LSL-hM3Dq* gene but did not harbor the *adipoq-Cre* transgene served as control animals. Adipocyte-specific Gα_q/11_−_/_− mice (adipo-G_q/11_ KO mice) were created by crossing *adipoq-Cre* mice with *Gα*_*q*_*flox/flox Gα*_*11*_^*−/−*^ mice^56^. The generation of *adipoq-CreER*^*T2*^*-AMPK β1* ^*flox/flox*^ *β2*^*flox/flox*^ mice has been described^37^. We also crossed IR^flox/flox^ mice (The Jackson Laboratory, stock # 006955) with adipo-GqD mice, resulting in *adipo-GqD IR*^*+/−*^ mice. In studies where *adipo-GqD IR*^*+/−*^ mice were used, LSL-GqD-IR^+/flox^ mice that do not carry the *adipoq-Cre* transgene served as control animals. All mouse strains were maintained on a C57BL/6 background.

All experiments were performed with adult male littermates, unless stated otherwise. Mice were housed at room temperature (23 °C; humidity: 30–40%) and provided with standard chow (7022 NIH-07 diet, 15% kcal fat, energy density 3.1 kcal/g; Envigo Inc.). Animals had free access to water and food and were kept on a 12-hr light/dark cycle. To induce obesity, 6–8-week-old male mice were switched to a high fat diet (HFD) (F3282, 60% kcal fat, energy density 5.5 kcal/g; Bio-Serv). Mice were maintained on the HFD for at least 8 weeks before initiating metabolic studies. All animal experiments were carried out according to the U.S. National Institutes of Health Guidelines for Animal Research and were approved by the National Institute of Diabetes and Digestive and Kidney Diseases Institutional Animal Care and Use Committee.

### Body composition

The lean and fat mass composition of mutant and control mice was measured using the 3-in-1 Echo MRI Composition Analyzer (Echo Medical System).

### In vivo metabolic tests

Male mice consuming regular chow (RC) or HFD were subjected to a series of in vivo metabolic tests. Adipo-GqD mice and control littermates that had been fasted for 4 hr received a single injection of CNO (10 mg/kg i.p.). Blood was collected from the tail vein just prior to injections (time ‘0’) and at specific postinjection time points. Adipo-GqD-IR^+/−^ mutant mice and their corresponding control mice received CNO (10 mg/kg i.p.). or saline after a 4 h fast, followed by the collection of blood samples at specific time points. Blood glucose levels were measured using a portable glucometer (Contour Glucometer, Bayer). Plasma samples were obtained by centrifuging blood samples at 4 °C for 10 min at ~12,000x *g*. Plasma insulin levels were measured using an ELISA kit (Crystal Chem Inc.), according to the manufacturer’s instructions. Plasma glycerol and FFA levels were determined using commercially available kits (Sigma-Aldrich). Plasma leptin levels were measured via ELISA (kit from R&D Systems).

For GTT studies, mice were fasted overnight for 16 h (unless stated otherwise) and then injected i.p. with a glucose bolus (RC mice: 2 g/kg; HFD mice: 1 g/kg) in the presence or absence of CNO (10 mg/kg i.p.). Blood glucose levels were determined at 0, 15, 30, 60, 90, and 120 min. For ITT studies, mice were fasted for 4 h and then injected i.p. with human insulin (0.75 or 1 U/kg: Humulin, Eli Lilly) in the absence or presence of CNO (10 mg/kg i.p.). Blood glucose levels were monitored at time ‘0’ and at defined post-injection time points. To study GSIS, adipo-GqD and control mice were fasted 4 h and then injected i.p. with glucose (1 or 2 g/kg) in the absence or presence of CNO (10 mg/kg i.p.). Plasma insulin levels were measured at defined post-injection time points via ELISA.

### Western blotting studies

Adipocytes/adipose tissues were lysed, and proteins were extracted using radioimmunoprecipitation assay (RIPA) buffer supplemented with cOmplete EDTA-free protease inhibitor cocktail (Sigma-Aldrich). Cell lysates were centrifuged at 12,000x *g* for 10 min, and protein concentrations were determined using a BCA protein assay kit (Pierce). Subsequently, proteins samples were denatured at 95 °C using NuPAGE LDS sample buffer (Thermo Fisher Scientific), and proteins were separated via 4–12% SDS–polyacrylamide gel electrophoresis. Proteins were then transferred to nitrocellulose membranes and incubated overnight at 4 °C with primary antibody. On the next day, membranes were washed thoroughly and then incubated with HRP-conjugated anti-rabbit or anti-goat secondary antibody, followed by visualization of separated protein bands using SuperSignal West Pico Chemiluminescent Substrate (Thermo Fisher Scientific) on the c600 Imaging System Imager (Azure Biosystems). Immunoreactive bands were quantified using Image J Software (NIH). Uncropped images are provided in the Source Data file.

The following antibodies were used (source, catalog #, if applicable, and dilution are indicated): AMPKα (Cell Signaling Technology, #2532, 1:1000), AMPKβ 1/2 (Dr. Gregory Steinberg, 1:1000), phospho-AMPKα (Thr172) (40H9) (Cell Signaling Technology, #2535, 1:1000), AS160 (Abcam, #134749, 1:2,000), phospho-AS160 (Thr642) (D27E6) (Cell Signaling Technology, #8881, 1:1000), HSL (Cell Signaling Technology, #4107, 1:1000), phospho-HSL (Ser565) (Cell Signaling Technology, #4137, 1:1,000), Glut4 (1F8) (Cell Signaling Technology, #2213, 1:1000), Na,K-ATPase (Cell Signaling Technology, #3010, 1:1000), HA-tag (C29F4) (Cell Signaling Technology, #3724, 1:1000), β-actin (13E5) (Cell Signaling Technology, #4970, 1:1000), anti-mouse IgG, HRP-linked antibody (Cell Signaling Technology #7076, 1:2000), antirabbit IgG, HRP-linked antibody (Cell Signaling Technology #7074, 1:2000).

### qRT-PCR analysis of gene expression

Total RNA was extracted from frozen tissues or cultured cells using the RNeasy mini kit (Qiagen), and SuperScript III First-Strand Synthesis SuperMix (Invitrogen) was used to prepare cDNA. The SYBR green method (Applied Biosystems) was used to perform quantitative PCR studies^57^. Gene expression data were normalized relative to the expression of 18 S rRNA using the ΔΔCt method. All PCR primers used in this study are listed in Supplementary Table 3.

### Differentiation of 3T3F442A cells

Mouse 3T3F442A cells (Kerafast, catalog # EF3002) were incubated with Dulbecco’s minimum essential medium (DMEM) containing 10% bovine calf serum (BCS) and 1% pen/strep in a 10% CO_2_ incubator at 37 °C. Cells were grown in 12-well collagen-coated plates. Once the cells had reached confluence, they were differentiated into mature adipocytes by a two-day incubation with DMEM containing 10% BCS, insulin (0.5 μg/ml), IBMX (250 μM), dexamethasone (0.5 μM), indomethacin (60 μM), and rosiglitazone (2 μM). After this treatment, cells were grown in DMEM containing 10% BCS and insulin (0.5 μM) for 48 hr. Subsequently, cells were maintained in DMEM containing 10% BCS and used for experiments.

### siRNA-mediated knockdown of AS160 and HSL expression in 3T3F442A cells

AS160 siRNA (mouse *Tbc1d4* siRNA) was purchased from Santa Cruz. HSL siRNA (*Lipe* smart pool mouse siRNA) and scrambled control siRNA were obtained from Dharmacon. On day 0, 3T3F442A cells were transfected with siRNA using Lipofectamine RNAiMAX (Thermo Fisher Scientific) by following the manufacturer’s instructions. Four hr later, cells were changed to differentiation media. On day 5, differentiated 3T3F442A adipocytes were used for further experiments.

### Isolation and differentiation of mouse preadipocytes

Mouse preadipocytes were isolated and differentiated using a standard protocol^40^. In brief, inguinal WAT (iWAT) was isolated from 8–10-week-old male mice and digested using collagenase 1 (Sigma–Aldrich) in Krebs-Ringer Hepes bicarbonate buffer (KRH buffer: 120 mM NaCl, 4 mM KH_2_PO_4_, 1 mM MgSO_4_, 1 mM CaCl_2_, 10 mM NaHCO_3_, and 30 mM HEPES; pH = 7.4). Digested tissue was passed through a 10 µm cell strainer and centrifuged at 300x *g* for 5 min at room temperature. The resulting cell pellet was resuspended and then filtered through a 40 µm cell strainer, and the resultant cell suspension was centrifuged (same conditions as above). Finally, the cell pellet was resuspended in DMEM containing 10% fetal bovine serum (FBS), supplemented with 1% pen/strep. Cells were grown in 12-well collagen-coated plates. Once the preadipocytes reached 100% confluency, they were differentiated into mature adipocytes by a two-day incubation with differentiation media containing insulin, IBMX, rosiglitazone, dexamethasone, and indomethacin (for details, see the previous paragraph). After this period, cells were incubated with DMEM containing 10% FBS, 0.5 μM insulin, and, when required, 100 nM 4-hydroxytamoxifen^37^ for another 48 h. Differentiated mouse adipocytes were then used for functional experiments.

### Preparation of differentiated human white adipocytes

Immortalized human white preadipocytes (hWAT-SVF cells; kindly provided by Dr. Yu-Hua Tseng, Joslin Diabetes Center, Boston, MA)^34^ were plated in collagen-coated 6- or 12-well plates and maintained in DMEM supplemented with 10% FBS and 1% pen/strep in a 5% CO_2_ incubator at 37 °C. Once cells reached 100% confluency, they were treated with induction media containing human insulin (0.5 μM), biotin (33 μM), pantothenate (17 μM), dexamethasone (0.1 μM), IBMX (500 μM), indomethacin (30 μM), and T3 (2 nM) for 28 days. Once the differentiation process was complete, media were replaced with DMEM containing 10% FBS.

### Adenoviruses

The adenovirus coding for hM3Dq (short name: GqD) (titer: 1.2 × 10^12^ transducing units/ml) was generated by the University of North Carolina at Chapel Hill Vector Core (Director: Dr. David Dismuke). The hM3Dq coding sequence^18^ was inserted into the pShuttle-CMV-IRES-mCitrine vector. The adenovirus coding for GFP (titer: 3.2 × 10^11^ IFU/mL) was a gift from the laboratory of Dr. Marc Montminy (Salk Institute for Biological Studies, La Jolla, CA). Adenoviruses were added to the growth medium at a multiplicity of infection of 50 MOI one day before the end of the adipocyte differentiation step (4 days after the start of differentiation). On the following day, the medium was replaced with fresh one. Assays were performed two days after the addition of viruses.

### In vivo tissue glucose uptake assay

Adipo-GqD mice and control littermates maintained on regular chow were subjected to in vivo tissue glucose uptake studies. In brief, mice were fasted overnight for 16 h and then injected i.p. with either saline or CNO (10 mg/kg) containing a trace amount of ^14^C-2-deoxyglucose (10 μCi; PerkinElmer). Forty minutes after injection, mice were euthanized, and multiple metabolically active tissues were collected, weighed, and homogenized. Radioactivity was measured as described^58^.

### Adipocyte glucose uptake assay

In vitro glucose uptake by adipocytes was determined following a published protocol^14^. Cells were grown in 12-well collagen-coated plates. On the day of experiment, differentiated adipocytes prepared from 3T3F442A cells or mouse pre-adipocytes were serum-starved for 3 hr and then washed with HBS buffer of the following composition (in mM): 20 HEPES [pH 7.4], 2.5 MgSO_4_, 1 CaCl_2_, 140 NaCl, and 5 KCl. Adipocytes were then incubated with various pharmacological agents as described in the text. Subsequently, cells were incubated with HBS buffer containing 0.5 μCi/ml ^3^H-2-deoxy-glucose (PerkinElmer) and 100 μM 2-deoxy-D-glucose (Sigma). Nonspecific glucose uptake was measured in the presence of 10 μM cytochalasin B (Sigma-Aldrich). Finally, stop solution (0.9% NaCl solution containing 25 mM glucose) was added to terminate glucose uptake. Adipocytes were then lysed with 0.05 N NaOH, and radioactivity was measured with a PerkinElmer liquid scintillation counter. Protein concentrations were determined by using the Bio-Rad Bradford assay kit. Glucose uptake was calculated as uptake of ^3^H-2-deoxy-glucose (in pmoles) per mg of protein (pmoles/mg/min).

### In vitro lipolysis assay

Differentiated 3T3F442A cells, hWAT cells, and mouse primary adipocytes were used for lipolysis measurements. Cells were grown in 12-well collagen-coated plates. Cells were serum-starved with DMEM basal media for 2 h, and then incubated with various pharmacological agents dissolved in DMEM for 3 h at 37 °C, as indicated in the text. Subsequently, the amount of glycerol released into the medium was determined as a measure of lipolysis. Cells were lysed using RIPA buffer to measure protein concentrations. Lipolysis data were expressed as the amount of glycerol released per mg of cellular protein. In the case of hWAT cells, KRB buffer (composition in mM: 135 NaCl, 5 KCl, 1 MgSO_4_, 0.4 K_2_HPO_4_, 20 HEPES, 1 CaCl_2_, 5 glucose, and 4% BSA; pH=7.4) served as assay buffer.

### GLUT4 translocation measured with differentiated 3T3-F442A cells

3T3-F442A cells were grown in 6-well collagen-coated plates. The cells were infected with GqD- or GFP-encoding adenoviruses (50 MOI) one day before the end of the differentiation step (4 days after the start of differentiation). After the addition of new media, the cells were incubated for 48 h with a GLUT4-myc-expressing adenovirus (50 MOI) (a kind gift by Dr. Susanna Keller, University of Virginia, Charlottesville, VA) 5 days after the start of the differentiation step. Subsequently, cells were incubated with CNO (10 μM) for 30 min, and plasma membranes were prepared as described using a standard protocol^59^. In brief, cells were homogenized in a buffer containing 250 mM sucrose, 10 mM Tris HCl, and 1 mM EDTA. Homogenates were centrifuged at 960 x *g* for 2 min at 4 °C. The resultant supernatant was centrifuged at 20,000 x *g* for 20 min at 4 °C. The pellet was resuspended in buffer and centrifuged at 160,000 x *g* for 60 min. Subsequently, the pellet was resuspended and re-centrifuged at 150,000 x *g* for 60 min. The amount of GLUT4 present in the resulting plasma membrane pellet was quantitated via western blotting using a commercially available mouse monoclonal anti-GLUT4 antibody (Cell Signaling Technology, #2213).

### CAMKK2 assay

Differentiated 3T3F442A cells infected with the GqD adenovirus (GqD-3T3F442A cells) were used to measure CAMKK2 activity. Cells were grown in 12-well collagen-coated plates. On the day of the experiment, GqD-3T3F442A cells were serum starved for 3 h and then treated with CNO (10 μM) or CNO (10 μM) plus STO-609 (20 μM) for 30 min. Subsequently, the cells were lysed, and CAMKK2 activity was measured using the CycLex® CaMKKβ Kinase Assay Kit (MBL International Corporation), following the manufacturer’s instructions.

### Treatment of mice with a selective CysLT_2_ receptor agonist

Adipo-GqD-IR ^+/−^ mutant mice and control littermates lacking the *adipoq-Cre* transgene that had been maintained on HFD for 12 weeks were injected with N-methyl LTC_4_ (NMLTC_4_; 10 μg/kg i.p.), a hydrolysis-resistant, selective CysLT_2_ receptor agonist^41^, followed by the measurement of blood glucose and plasma FFA levels. In glucose tolerance testes, MLTC_4_ (10 μg/kg) was co-injected i.p. with 1 g/kg of glucose.

### Ex vivo IP_1_ assay with mouse adipose tissues

Twelve male WT C57BL/6 mice (age: 8 weeks; Taconic) were injected with LiCl (10 mmoles/kg, s.c.) prior to the beginning of the dark cycle (6 pm). Six mice had free access to food, while the remaining six mice were fasted overnight. On the next morning (8 am), the mice were euthanized, and adipose depots (iWAT and eWAT) were isolated and quickly frozen on dry ice. Adipose tissues were homogenized in RIPA buffer, supplemented with LiCl (50 mM) and complete EDTA-free protease inhibitor cocktail (Roche). Briefly, 100–200 mg tissue was incubated in RIPA buffer with LiCl (50 mM) for 15 min on ice, followed by homogenization in lysing matrix E tubes using a Precellys® 24 homogenizer. The resultant supernatant was subjected to centrifugation (6000 x *g*) for 15 min to separate the ‘fat cake’ (white lipid layer) from the supernatant containing the loose pellet. The supernatant and the loose pellet (cell debris) were subjected to an additional centrifugation step (1200 x *g*, 15 min) to yield the protein lysate (supernatant). IP_1_ levels in the protein lysates were measured using the IP-one ELISA kit from Cisbio following the manufacturer’s instructions.

### Source of human subcutaneous adipose tissue

We complied with all relevant ethical regulations for work with human participants. Subjects were admitted to the Metabolic Clinical Research Unit in the Hatfield Clinical Research Center of the National Institutes of Health (Bethesda, MD) to participate in an NIDDK/NIAMS institutional review board-approved protocol (ClinicalTrials.gov identifier NCT00428987), after having given informed consent. The human adipose tissue samples analyzed in this study have been described in detail in a recent study^40^. Human subcutaneous fat tissue samples were obtained from the abdominal area under local anesthesia using an aspiration needle. After the isolation of mRNA, gene expression levels were determined via qRT-PCR.

### Statistics

Data were collected and analyzed by using Prism 8 (GraphPad) and Microsoft Excel software. All data are expressed as means ± s.e.m. for the indicated number of observations. Prior to performing specific statistical tests, we performed tests for normality and homogeneity of variance. Data were then tested for statistical significance by one- or two-way ANOVA, followed by the indicated post hoc tests, or by using a two-tailed unpaired Student’s *t* test, as appropriate. Correlation analysis was conducted by Spearman’s test. A *p* value of <0.05 was considered statistically significant. The specific statistical tests that were used are indicated in the figure legends.

### Reporting summary

Further information on research design is available in the Nature Research Reporting Summary linked to this article.

## Supplementary information

Supplementary Information

Reporting Summary

## Data Availability

All data generated or analyzed during this study are included in this published article (and its supplementary information files). Source data are provided as a Source Data file. Source data are provided with this paper.

## Source data

Source Data
