# Supplementary material for: Adipocyte Gq signaling is a regulator of glucose and lipid homeostasis in mice
Source: Nat Commun. 2022 Mar 29;13:1652. doi: 10.1038/s41467-022-29231-6 (PMC8964770; doi:10.1038/s41467-022-29231-6)
Supplement: Supplementary file 1 — Supplementary Information [file 41467_2022_29231_MOESM1_ESM.pdf]

Supplemental Information

Adipocyte G_q signaling is a regulator of glucose and lipid homeostasis in mice

Takefumi Kimura[1], Sai P. Pydi[1], Lei Wang[1], Dhanush Haspula[1], Yinghong Cui[1], Huiyan Lu[2], Gabriele M. König[3], Evi Kostenis[4], Gregory R. Steinberg[5], Oksana Gavrilova[6], Jürgen Wess[1]

[1]Molecular Signaling Section, Laboratory of Bioorganic Chemistry, National Institute of Diabetes and Digestive and Kidney Diseases, Bethesda, MD 20892, USA
[2]Mouse Transgenic Core Facility, National Institute of Diabetes and Digestive and Kidney Diseases, Bethesda, Maryland 20892, USA
[3]Institute of Pharmaceutical Biology, University of Bonn, 53115 Bonn, Germany
[4]Molecular, Cellular and Pharmacobiology Section, Institute of Pharmaceutical Biology, University of Bonn, 53115 Bonn, Germany
[5]Department of Medicine and Department of Biochemistry and Biomedical Sciences, McMaster University, Hamilton, ON L8K 4P1, Canada, McMaster University, Hamilton, ON L8K 4P1, Canada
[6]Mouse Metabolism Core National Institute of Diabetes and Digestive and Kidney Diseases, Bethesda, MD 20892, USA

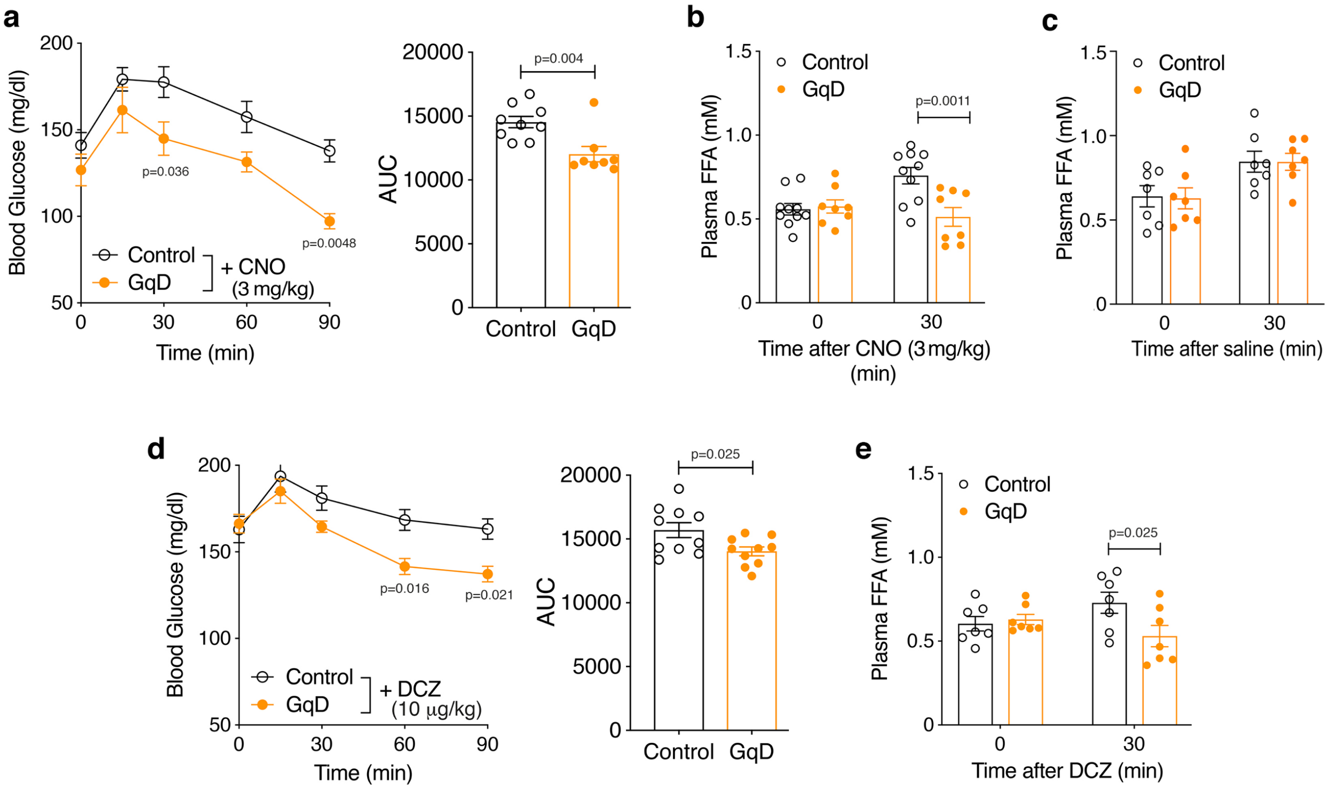

Supplementary Fig. 1. Acute treatment of adipo-GqD mice with CNO (3 mg/kg) or DCZ (10 µg/kg). a-c, Blood glucose levels (**a**) and plasma free fatty acid (FFA) levels 30 min after injection of control and adipo-GqD mice (GqD) with CNO (3 mg/kg i.p.) (**b**) or saline (**c**). Mice were fasted for 4 hr prior to injections (n=7-10 per group). *LSL-hM3Dq* mice that did not harbor the *adipoq-Cre* transgene served as control animals. **d**, **e,** Blood glucose levels (**d**) and plasma FFA levels (**e**) 30 min after injection of control and adipo-GqD mice with deschloroclozapine (DCZ) (10 µg/kg i.p.). Mice were subjected to a 4 hr fast prior to DCZ administration (n=7-10 per group). All studies were carried out with male mice that were 10-16 weeks old. Data are presented as means ± s.e.m. (two tailed Student's t-test). Source data are provided as a Source data file.

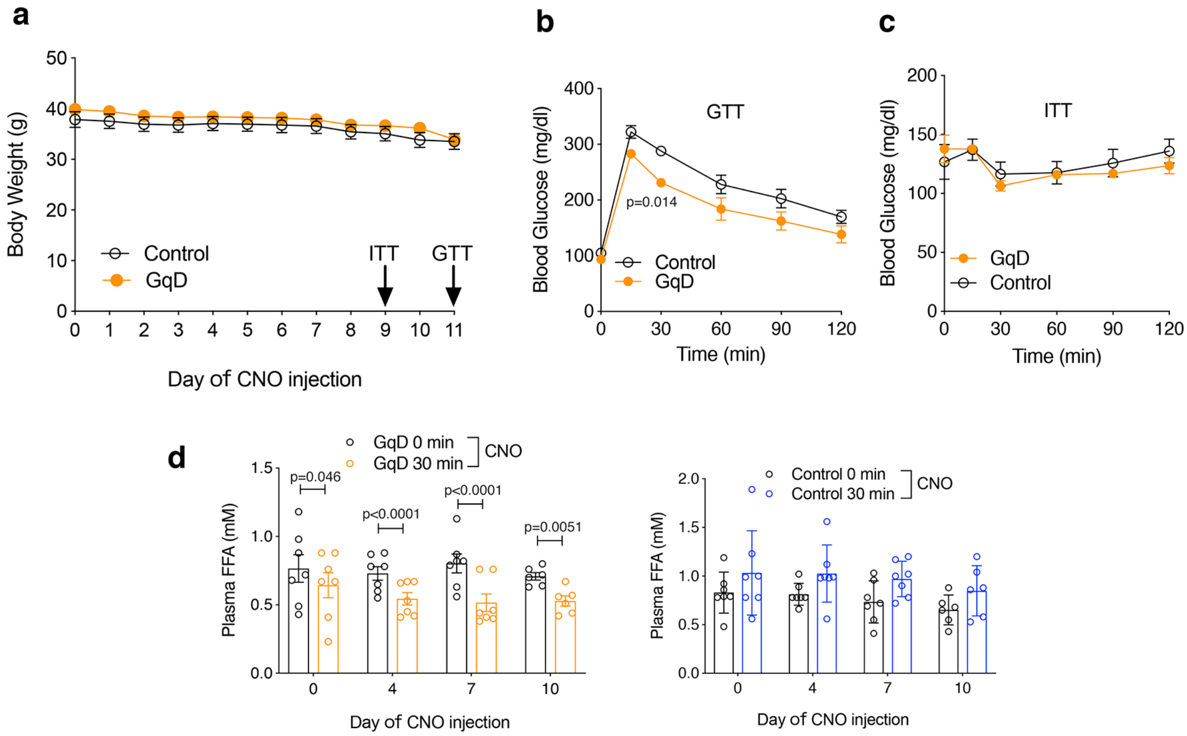

Supplementary Fig. 2. Chronic activation of adipocyte G_q signaling improves glucose homeostasis. a, Chronic CNO treatment of adipo-GqD mice (GqD) does not affect body weight. Adipo-GqD mice and control mice consuming regular chow received daily i.p. injections of CNO (10 mg/kg) for 11 days (n=6 or 7; 20-week-old males). **b**, I.p. glucose tolerance test (GTT) after chronic CNO treatment of mice for 11 days (2 g/kg glucose). **c**, Insulin tolerance test (ITT) after chronic CNO treatment for 9 days (1 U/kg insulin, i.p.). **d**, Plasma free fatty acid (FFA) levels during chronic CNO treatment of adipo-GqD mice and control littermates. Plasma FFA levels were determined just prior to and 30 min after CNO injections. Data are given as means ± s.e.m. **b**: two-way ANOVA followed by Bonferroni's post-hoc test; **d**: two-tailed Student's t test**.** Source data are provided as a Source data file.

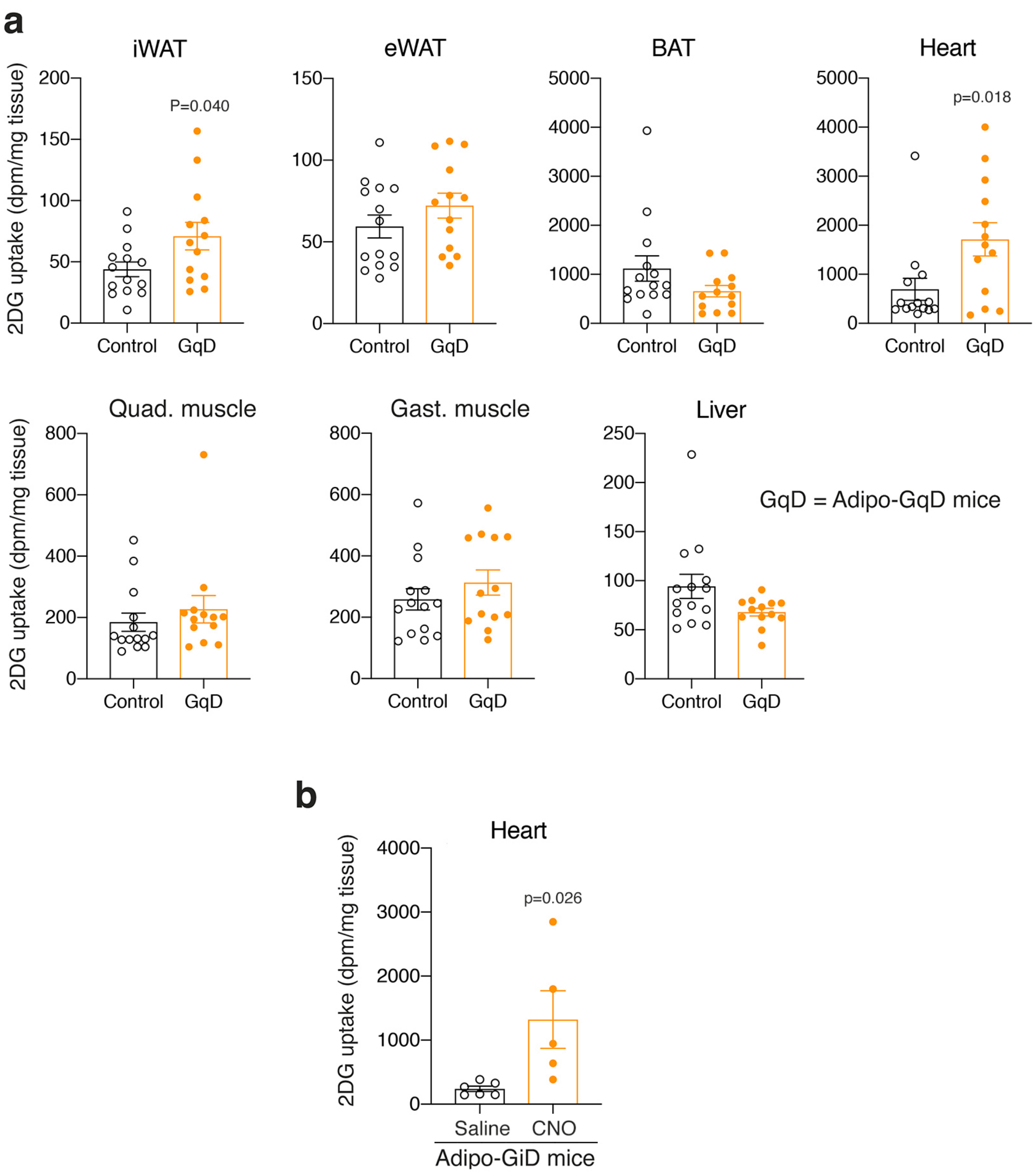

Supplementary Fig. 3. CNO treatment of adipo-GqD mice promotes glucose uptake by iWAT and heart. a, In vivo 2-deoxy-D-glucose uptake (2-DG) uptake studies were performed with adipo-GqD and control mice maintained on regular chow diet (n=13 or 14 per group). *LSL-hM3Dq* mice that did not harbor the *adipoq-Cre* transgene served as control animals. Mice were fasted overnight and then injected with CNO (10 mg/kg i.p.) and a trace amount of ^{14}C-2-DG. Forty min later, the mice were euthanized, and the ^{14}C-2-DG content of various peripheral tissues

was determined. **b,** CNO stimulates cardiac ^{14}C-2-DG uptake in adipo-GiD mice expressing a G_i-coupled DREADD selectively in adipocytes[1]. Adipo-GiD mice were injected with either saline or CNO (10 mg/kg i.p.) (n=5 or 6 per group). Cardiac ^{14}C-2-DG uptake was determined as described in (**a**). For all experiments, 14-20-week-old male mice were used. See Methods for experimental details. Data are presented as means ± s.e.m. (two-tailed Student's t-test). Source data are provided as a Source data file. iWAT, inguinal white adipose tissue; eWAT, epididymal white adipose tissue; BAT, brown adipose tissue.

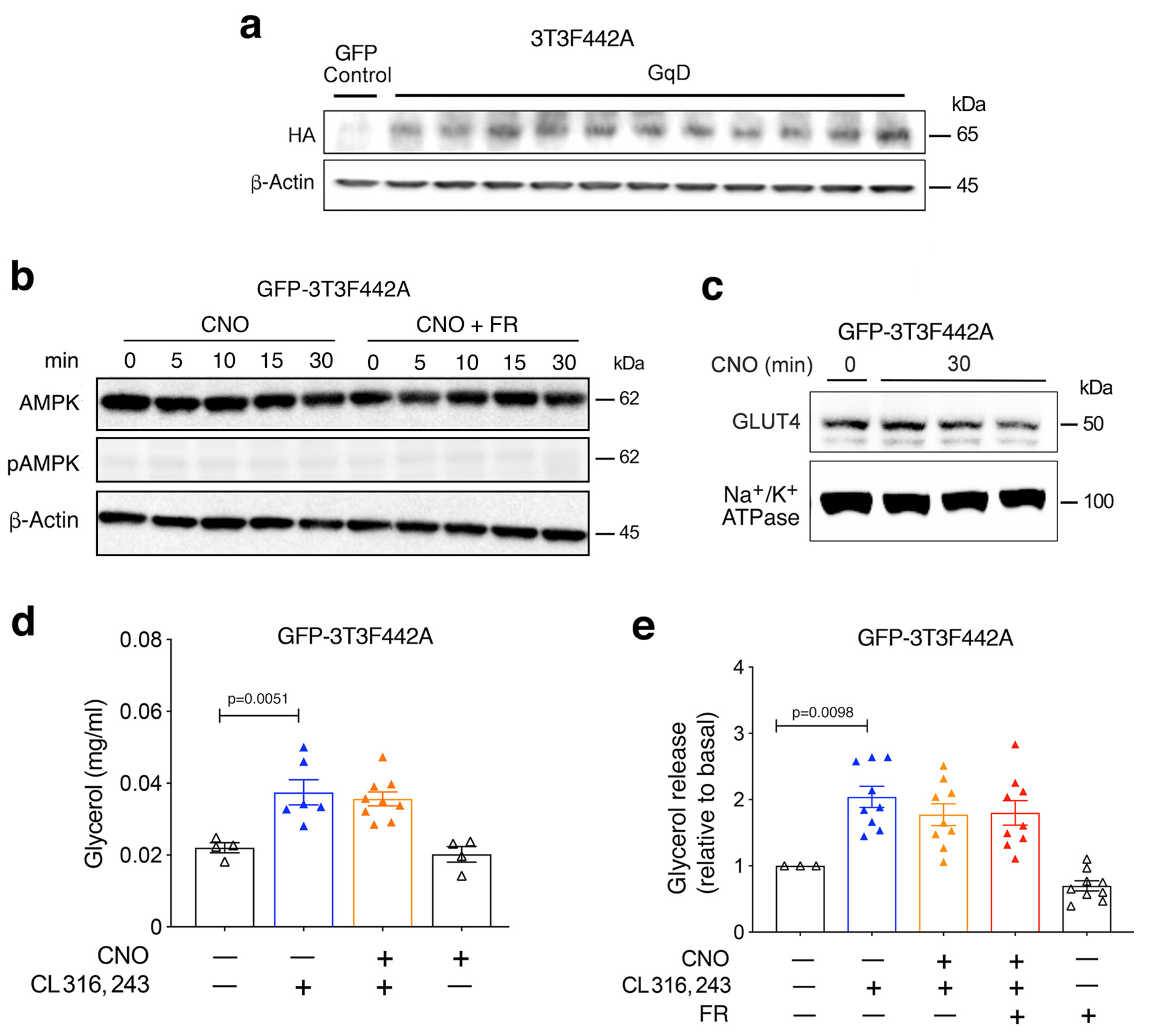

Supplementary Fig. 4. Expression of GqD in 3T3F442A cells and studies with GFP-3T3F442A control cells. a, Western blot analysis of differentiated 3T3F442A cells infected with an adenovirus coding for GqD (GqD-3T3F442A cells). The GqD protein was detected by an anti-HA antibody directed against the HA epitope tag fused to the N-terminus of GqD. Control cells were infected with an adenovirus coding for GFP (GFP-3T3F442A cells). **b,** Western blot analysis of GFP-3T3F442A control cells after treatment with CNO (10 μM) or CNO (10 μM) plus FR900359 (FR; $G_{q/11}$ inhibitor; 1 μM). Blots were probed with antibodies directed against total AMPK or pAMPK (T172). **c,** Western blot analysis of the plasma membrane fraction of GFP-3T3F442A control cells. Cells were treated with CNO (10 μM) for 30 min, followed by cell lysis and detection of GLUT4 expression via Western blotting. **d,** Glycerol release from GFP-

3T3F442A control cells after treatment with CL316,243 (β3-adrenergic receptor agonist, 100 nM) is similar in the presence or absence of CNO (10 μM). **e**, CL316,243 (100 nM)-induced glycerol release from GFP-3T3F442A cells remains unaffected by FR (1 μM) treatment. In (**e**), basal glycerol release measured in the absence of any drugs was set equal to 1 in each individual experiment. The experiments shown in (**a-c**) were independently repeated twice with similar results. Data are presented as means ± s.e.m. of at least three independent experiments (one-way ANOVA followed by Bonferroni's post-hoc test). Source data are provided as a Source data file.

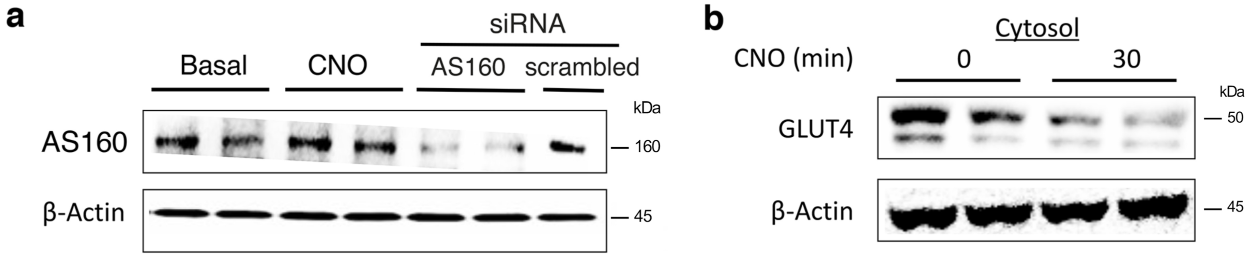

Supplementary Fig. 5. Studies with GqD-3T3F442A cells examining the specificity of the AS160 antibody and cytoplasmic GLUT4 levels. a, Specificity of the AS160 antibody used. GqD-3T3F442A cells were treated with CNO (10 μM) for 15 min, followed by the preparation of cell lysates and the detection of AS160 via Western blotting. To confirm the specificity of the AS160 antibody, GqD-3T3F442A cells were transfected with either AS160-specific siRNA or scrambled control siRNA. Cells transfected with AS160-specific siRNA show a very pronounced reduction of the AS160 immunoreactive signal, indicative of the specificity of the AS160 antibody used. **b**, Western blot analysis of cytosolic GLUT4. After treatment of GqD-3T3F442A cells with CNO (10 μM) for 30 min, the cytosolic fraction was isolated and subjected to immunoblotting analysis using an anti-GLUT4 antibody. The experiments shown in (**a, b**) were independently repeated twice with similar results.

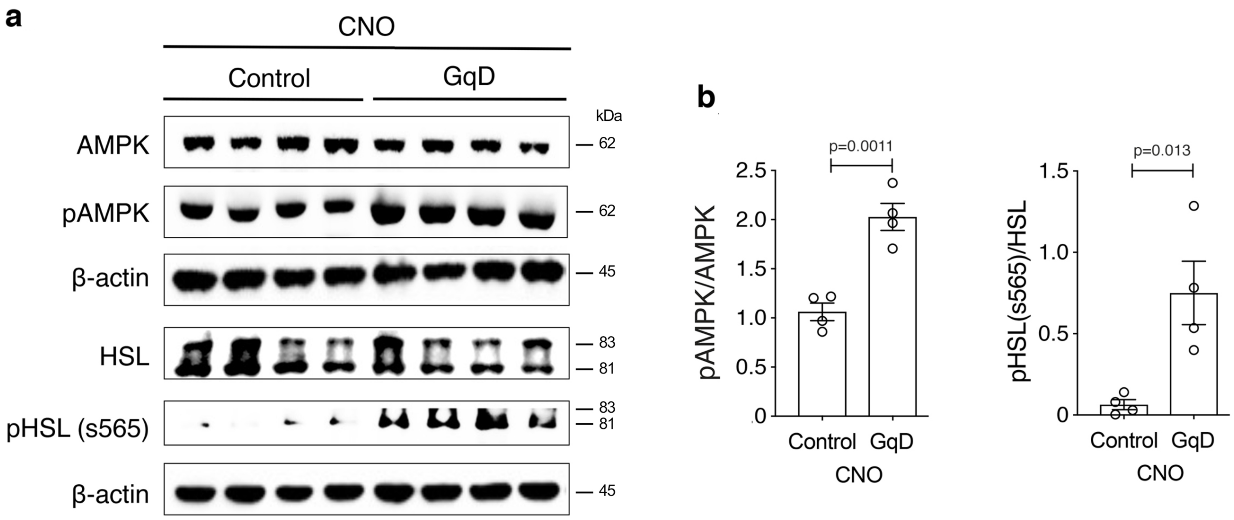

Supplementary Fig. 6. Activation of adipocyte G_q signaling in vivo stimulates the formation of pAMPK and pHSL(S565) in adipose tissue. Adipo-GqD (GqD) and control mice were injected with CNO (10 mg/kg i.p.) (n=4 per group; 12-week-old males). Fifteen min later, inguinal WAT (iWAT) was collected and subjected to Western blotting studies using the indicated antibodies. *LSL-hM3Dq* mice that did not harbor the *adipoq-Cre* transgene served as control animals. **b**, Quantitative analysis of pAMPK/AMPK and pHSL(S565)/HSL protein expression levels shown in (**a**). Data are presented as means ± s.e.m. (two tailed Student's t-test). Source data are provided as a Source data file.

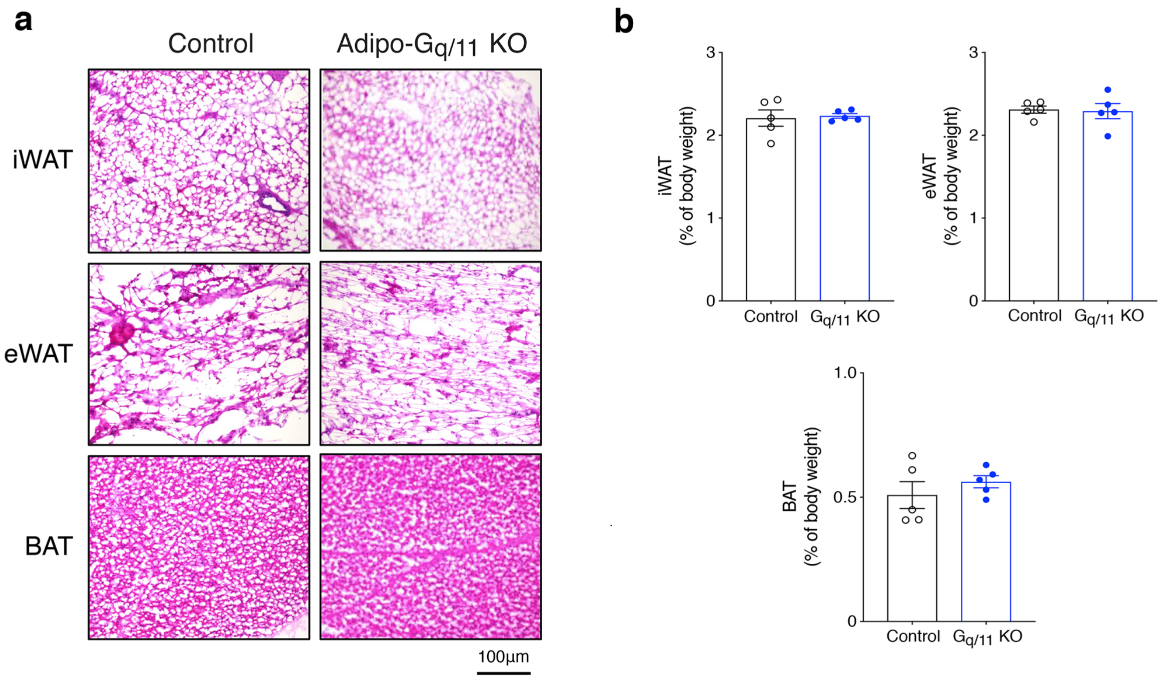

Supplementary Fig. 7. Analysis of adipose tissues from adipo-$G_{q/11}$ KO mice. a, H&E staining of iWAT, eWAT, and BAT from adipo-$G_{q/11}$ KO mice and control littermates (*Gα*$_q$*flox/flox Gα*$_{11}$−/− mice lacking the *adipoq-Cre* transgene). This experiment was carried out with three mice per group, yielding similar results. **b,** Adipose tissue weight expressed as % of body weight. Data are presented as means ± s.e.m. (n=5 per group; 12-week-old males). iWAT, inguinal white adipose tissue; eWAT, epididymal white adipose tissue; BAT, brown adipose tissue.

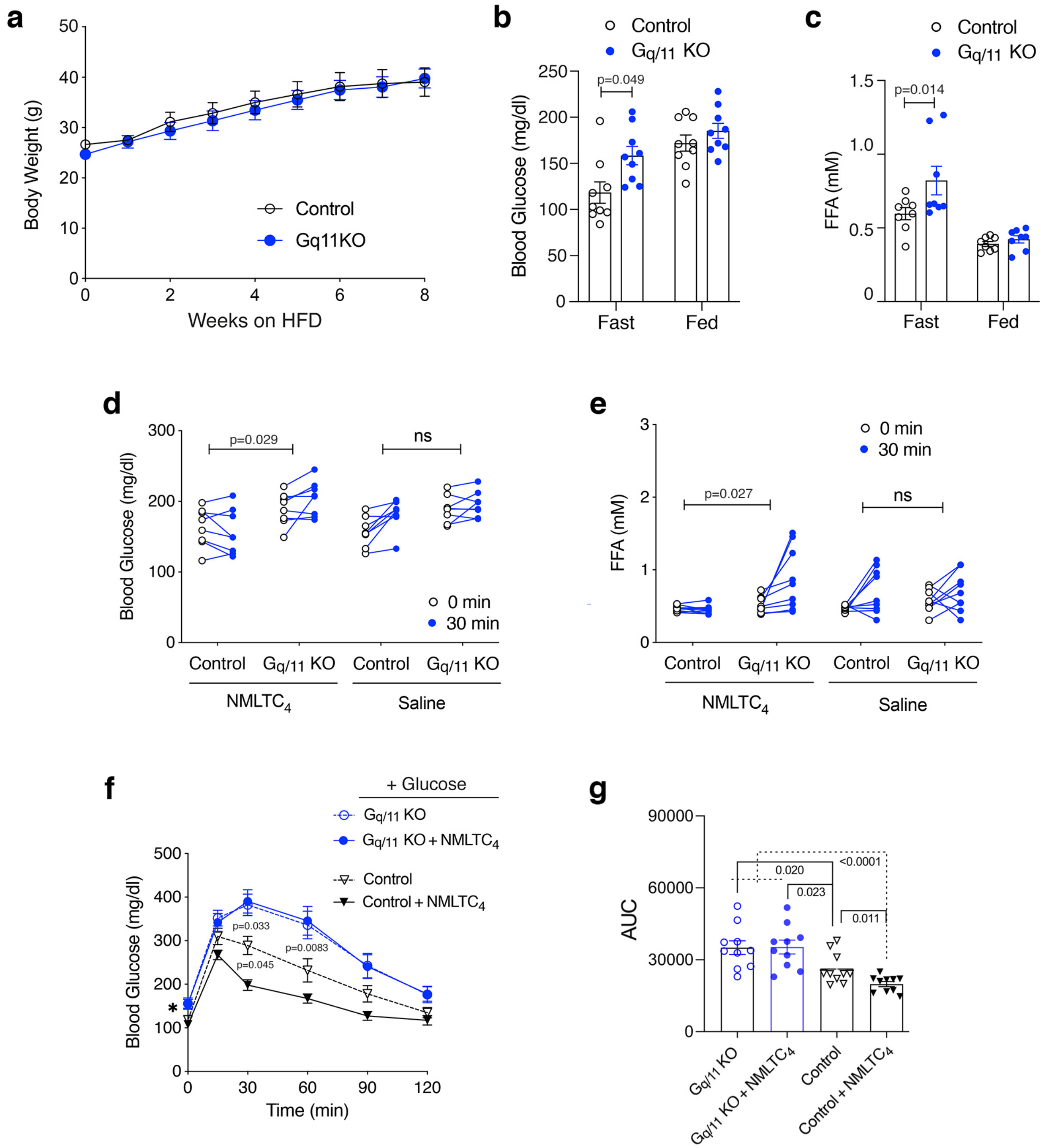

Supplementary Fig. 8. The beneficial metabolic effects of NMLTC4 in obese mice require the presence of adipocyte $G_{q/11}$. **a,** Changes in body weight of adipo-$G_{q/11}$ KO mice and control littermates ((*Gαqflox/flox Gα11−/−* mice lacking the *adipoq-Cre* transgene) maintained on high fat diet (HFD) for 8 weeks (n=10). **b, c,** Blood glucose (**b**) and plasma FFA (**c**) levels of adipo-$G_{q/11}$ KO and control mice under fasting and fed conditions (n=8 or 9 per group). **d, e,** Blood glucose (**d**) and plasma free fatty acid (FFA) (**e**) levels of control and adipo-$G_{q/11}$ KO mice 30

min after injection of $NMLTC_4$ (10 μg/kg i.p.) or saline, following a 4 hr fast (n=9 per group). **f,** Glucose tolerance test (GTT; i.p.) performed with control and adipo-$G_{q/11}$ KO mice (n=10 per group) with or without $NMLTC_4$ treatment (10 μg/kg i.p.) after an overnight fast (16 hr). **g,** AUC values for the curves shown in panel (**f**). Metabolic tests **(b-f)** were carried out with male mice that were ~16 weeks old. Data are given as means ± s.e.m. **b, c, g**: two-tailed Student's t test; **d, e,** two-way ANOVA; **f**: two-way ANOVA followed by Bonferroni's post-hoc test; interaction effect between 'time' and 'group' (p=0.033 and p=0.0083: $G_{q/11}$ KO vs. control; p=0.045: control vs. control + $NMLTC_4$). ns, no statistically significant difference. Source data are provided as a Source data file.

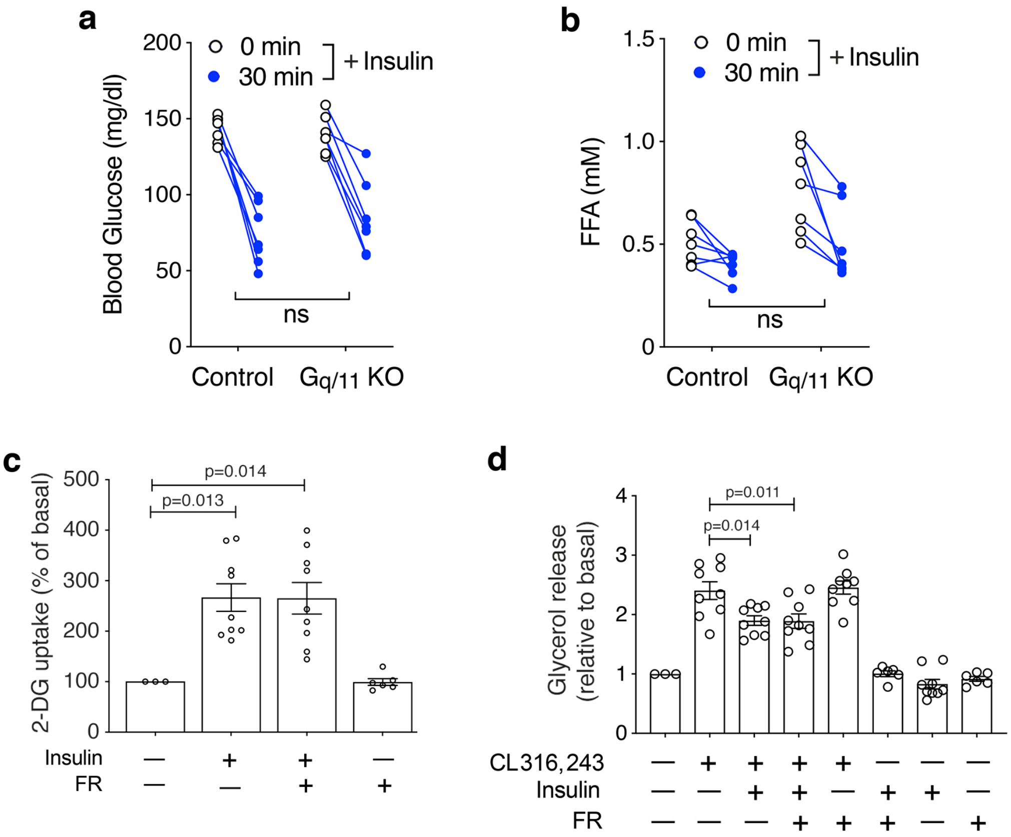

Supplementary Fig. 9. Lack of adipocyte $G_{q/11}$ signaling does not interfere with the glucose-lowering and antilipolytic actions of insulin in vivo and in vitro. **a, b,** Insulin treatment of adipo-$G_{q/11}$ KO and control mice. Adipo-$G_{q/11}$ KO mice and control littermates (*G*α_q*flox/flox G*α_{11}−/− mice lacking the *adipoq-Cre* transgene) consuming regular chow were injected with insulin (0.75 U /kg i.p.) following a 4 hr fast (n=7 per group; 12-week-old males). Blood glucose levels (**a**) and plasma free fatty acid (FFA) levels (**b**) were measured immediately before (‘0’ min) and 30 min after insulin injection. **c,** Insulin-induced glucose uptake in 3T3F442A cells in the absence or presence of a $G_{q/11}$ inhibitor. Insulin (10 nM)-stimulated uptake of ^{14}C-2-Deoxy-D-glucose (2-DG) was studied in the presence or absence of FR900359 (FR; $G_{q/11}$ inhibitor; 1 μM).
d, Antilipolytic activity of insulin in 3T3F442A cells in the absence or presence of a $G_{q/11}$ inhibitor. Incubation of 3T3F442A cells with CL316,243 (100 nM), a selective β3-adrenergic receptor agonist, led to a robust increase in glycerol release. This effect was significantly reduced in the presence of insulin (10 nM). Co-incubation with FR900359 (FR; $G_{q/11}$ inhibitor; 1 μM) had no significant effect on the magnitude of the antilipolytic effect of insulin. In (**c**) and (**d**), data were normalized relative to values obtained in the absence of any drugs. Data are presented as means ± s.e.m. of three independent experiments (**c, d**). **a, b**: two-way ANOVA; interaction effect between ‘time’ and ‘group’; **c, d**: one-way ANOVA followed by Bonferroni’s post-hoc test. ns, no statistically significant difference. Source data are provided as a Source data file.

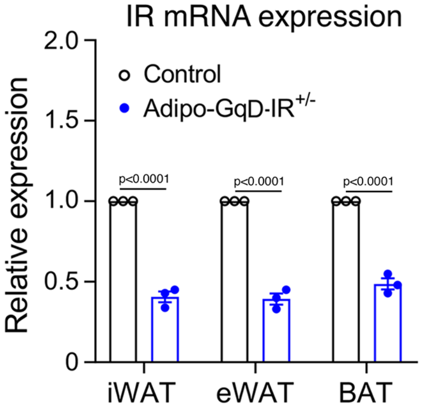

Supplementary Fig. 10. Insulin receptor mRNA expression in different adipose tissue depots. Relative expression of insulin receptor (IR) mRNA in adipose tissues of adipo-GqD-IR$^{+/-}$ mice and control littermates (LSL-GqD-IR$^{+/flox}$ mice that do not carry the *adipoq-Cre* transgene) (n=3 per group). Data were obtained via qRT-PCR, as described under Methods. IR transcript levels were expressed relative to mRNA levels obtained with adipose tissue RNA from control mice. Data are given as means ± s.e.m. **(**two-tailed Student's t test). Source data are provided as a Source data file. iWAT, inguinal white adipose tissue; eWAT, epididymal white adipose tissue; BAT; brown adipose tissue.

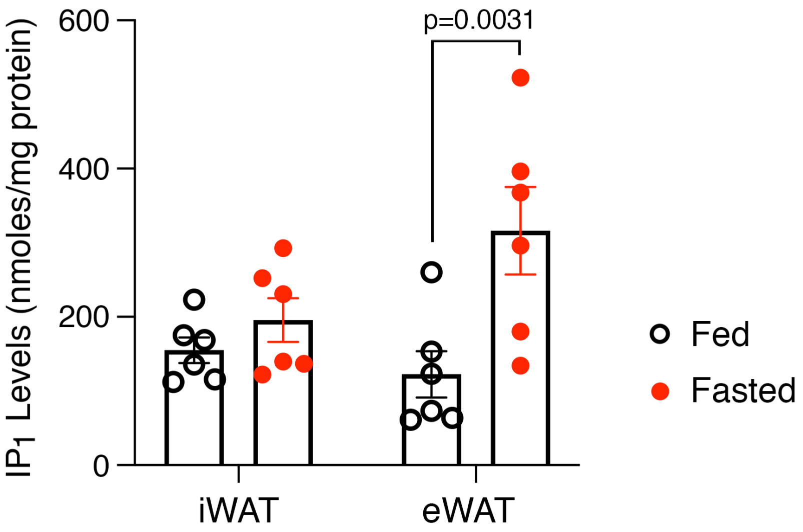

Supplementary Fig. 11. Fasting increases IP_1 levels in mouse adipose tissue (eWAT). Male WT C57BL/6 mice (age: 8 weeks; Taconic) maintained on regular chow were injected with LiCl (10 mmoles/kg, s.c.) prior to the beginning of the dark cycle (6 pm). One group of mice had free access to food (fed), while the other group was fasted overnight for 14 hr (fasted). On the next morning (8 am), adipose tissue lysates were prepared from iWAT and eWAT, followed by the determination of IP_1 levels. Data are given as means ± s.e.m. (n=6 mice per group; two-way ANOVA followed by Šidák correction post-hoc test). Source data are provided as a Source data file. iWAT, inguinal white adipose tissue; eWAT, epididymal white adipose tissue.

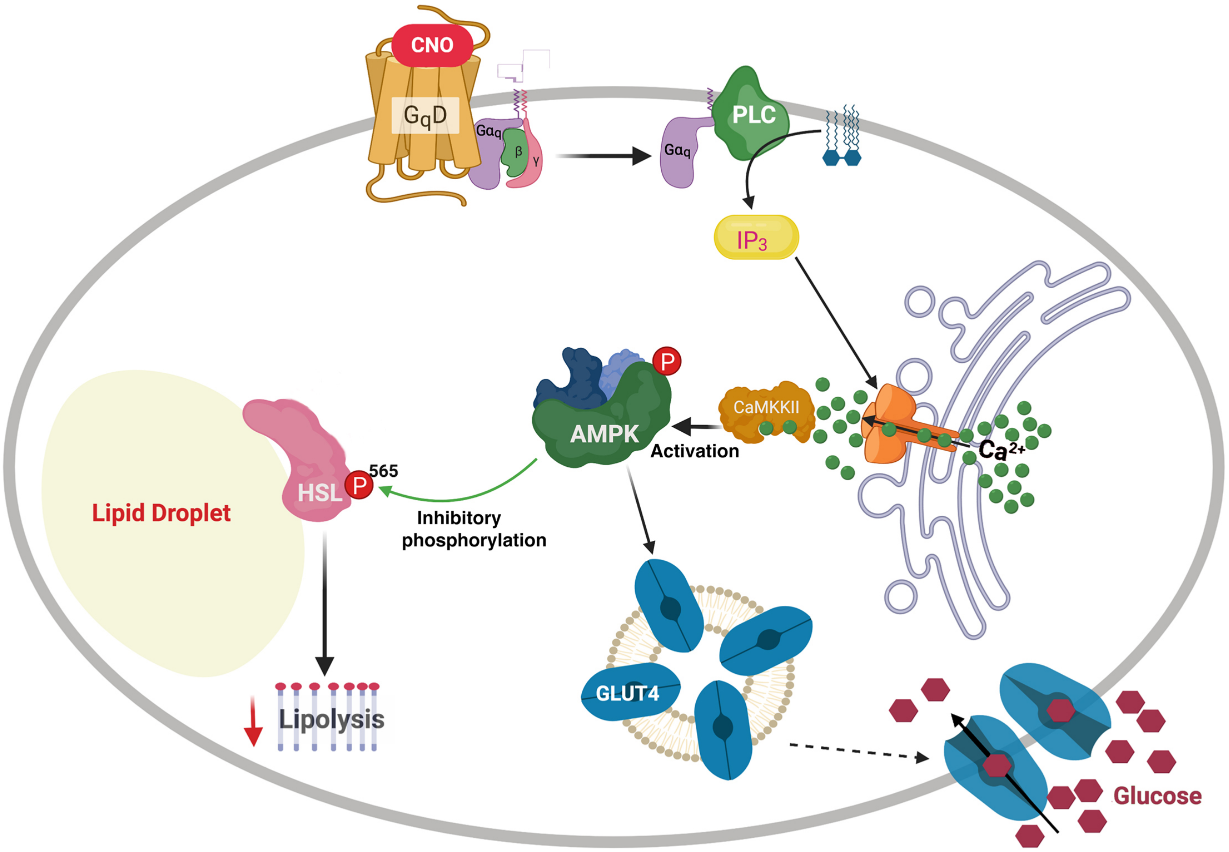

Supplementary Fig. 12. Scheme illustrating how receptor-mediated activation of G_q signaling in adipocytes enhances glucose uptake and inhibits lipolysis.

Supplementary Table 1. G_q-coupled receptors with relatively high expression in iWAT and eWAT of C57BL/6N mice, as determined via RNA-seq

iWAT					
	Gene	Encoded receptor	HFD/RC ratio	RPKM RC	RPKM HFD
	P2ry6	$P2Y_6$ receptor	20.284	0.341	6.911
	Htr2b	$5\text{-}HT_{2B}$ receptor	19.083	0.025	0.471
	C5ar1	Complement component 5a receptor 1	8.539	2.641	22.55
	Avpr1a	V_{1a} Vasopressin receptor	8.052	0.142	1.140
	Cysltr1	Cysteinyl leukotriene receptor 1	3.959	0.453	1.795
	Tbxa2r	Thromboxane receptor	3.098	0.089	0.276
	Cysltr2	Cysteinyl leukotriene receptor 2	2.456	0.109	0.269
	P2ry2	$P2Y_2$ receptor	3.774	1.882	7.101
	Gpr64	Orphan adhesion GPCR	0.273	2.325	0.634
	Kiss1r	Kisspeptin receptor	0.542	0.628	0.340

eWAT					
	Gene name	Encoded receptor	HFD/RC ratio	RPKM RC	RPKM HFD
	P2ry6	$P2Y_6$ receptor	23.686	1.214	28.762
	Prokr1	Prokineticin receptor 1	19.674	0.048	0.950
	C5ar1	Complement component 5a receptor 1	17.229	6.421	110.63
	Cysltr1	Cysteinyl leukotriene receptor 1	5.438	0.690	3.750
	Cysltr2	Cysteinyl leukotriene receptor 2	2.671	0.097	0.258
	Hrh1	Histamine H_1 receptor	2.250	0.166	0.373
	Gpr75	Class A orphan receptor	0.076	0.170	0.013
	Gpr64	Orphan adhesion GPCR	0.088	3.914	0.346
	Cckbr	Cholecystokinin B receptor	0.071	0.033	0.002
	Kiss1r	Kisspeptin receptor	0.197	0.938	0.185
	Ntsr2	Neurotensin receptor 2	0.249	2.922	0.728
	Ptgfr	Prostaglandin F receptor	0.323	1.752	0.566
	Ednra	Endothelin A receptor	0.490	3.373	1.651
	Lphn1	Latrophilin 1 (adhesion GPCR)	0.504	1.186	0.598
	Tbxa2r	Thromboxane receptor	0.513	0.217	0.111

The data shown in this table were extracted from RNA-seq data that we previously deposited under GEO accession numbers GSE131861 (RC data) and GSE134914 (HFD data), respectively[1]. RC, regular chow; HFD, high fat diet; iWAT, inguinal white adipose tissue; eWAT, epididymal white adipose tissue; RPKM, reads per kilo base per million mapped reads.

Supplementary Table 2. G_q-coupled receptors expressed at relatively high levels in human subcutaneous adipose tissues, as determined via qRT-PCR

Gene name	Encoded receptor	Abdominal fat (rel. expression)	Gluteofemoral fat (rel. expression)
LPHN1	Latrophilin 1 (adhesion GPCR)	0.154	0.151
PTAFR	Platelet-activating factor receptor	0.009	0.012
LPAR1	Lysophosphatidic acid receptor 1	0.025	0.016
LPAR2	Lysophosphatidic acid receptor 2	0.0032	0.0032
GPR75	Class A orphan receptor	0.0039	0.0047
CysLT2	Cysteinyl leukotriene receptor 2	0.0021	0.0021
GPR56	Orphan adhesion GPCR	0.079	0.083
FDZ5	Frizzled-5	0.022	0.022

The data shown in this table were extracted from a recently published article[2]. Only receptors for which gene expression data were quantitated are included in this list. Gene expression data are presented as mean expression relative to peptidylprolyl isomerase A (PPIA).

Supplementary Table 3. Primers used for q-RT-PCR studies

Gene	Species	Primer sequence (forward)	Primer sequence (reverse)
GqD (hm3Dq)	Human	5'-TCACAGCACCATCCTCAACT-3'	5'-GAAAACTGCCTCCATCGTCC-3'
Gnaq ($G\alpha_q$)	Mouse	5'-GTCGGGCTACTCTGACGAAGA-3'	5'-TGTGCATGAGCCTTATTGTGTTC-3'
Gna11($G\alpha_{11}$)	Mouse	5'-GACGCCTTCATCCGAGTCG-3'	5'-CGGCCTCTTTGTCAGCTTTAGA-3'
Insr (IR)	Mouse	5'-CCACCAATACGTCATTCACAAC-3'	5'-GGGCAGATGTCACAGAATCAA-3'
18S rRNA	Mouse	5'-CGGCTACCACATCCAAGGAA-3'	5'-GCTGGAATTACCGCGGCT-3'
CYSLTR2	Human	5'-CAGGGCAGCTGAAAGACAGA-3'	5'-TCTCCATACCTTGCATGGACC-3'
18S rRNA	Human	5'-GCAATTATTCCCCATGAACG-3'	5'-GGCCTCACTAAACCATCCAA-3'

References

1. Wang, L., *et al.* Adipocyte G(i) signaling is essential for maintaining whole-body glucose homeostasis and insulin sensitivity. *Nat Commun* **11**, 2995 (2020).
2. Amisten, S., *et al.* An atlas of G-protein coupled receptor expression and function in human subcutaneous adipose tissue. *Pharmacol Ther* **146**, 61-93 (2015).
