# Supplementary material for: Adipocyte Gq signaling is a regulator of glucose and lipid homeostasis in mice
Source: Nat Commun. 2022 Mar 29;13:1652. doi: 10.1038/s41467-022-29231-6 (PMC8964770; doi:10.1038/s41467-022-29231-6)
Supplement: Supplementary file 2 — Reporting Summary [file 41467_2022_29231_MOESM2_ESM.pdf]

nature research

Corresponding author(s): Jürgen Wess

Last updated by author(s): 03-03-2022

Reporting Summary

Nature Research wishes to improve the reproducibility of the work that we publish. This form provides structure for consistency and transparency in reporting. For further information on Nature Research policies, see Authors & Referees and the Editorial Policy Checklist.

Statistics

For all statistical analyses, confirm that the following items are present in the figure legend, table legend, main text, or Methods section.

n/a	Confirmed	
☐	☒	The exact sample size (*n*) for each experimental group/condition, given as a discrete number and unit of measurement
☐	☒	A statement on whether measurements were taken from distinct samples or whether the same sample was measured repeatedly
☐	☒	The statistical test(s) used AND whether they are one- or two-sided<br>*Only common tests should be described solely by name; describe more complex techniques in the Methods section.*
☐	☒	A description of all covariates tested
☐	☒	A description of any assumptions or corrections, such as tests of normality and adjustment for multiple comparisons
☐	☒	A full description of the statistical parameters including central tendency (e.g. means) or other basic estimates (e.g. regression coefficient) AND variation (e.g. standard deviation) or associated estimates of uncertainty (e.g. confidence intervals)
☐	☒	For null hypothesis testing, the test statistic (e.g. F, t, r) with confidence intervals, effect sizes, degrees of freedom and P value noted<br>*Give P values as exact values whenever suitable.*
☒	☐	For Bayesian analysis, information on the choice of priors and Markov chain Monte Carlo settings
☒	☐	For hierarchical and complex designs, identification of the appropriate level for tests and full reporting of outcomes
☒	☐	Estimates of effect sizes (e.g. Cohen's *d*, Pearson's *r*), indicating how they were calculated

Our web collection on statistics for biologists contains articles on many of the points above.

Software and code

Policy information about availability of computer code

Data collection	Prism9, Excel
Data analysis	Prism9, Excel, ImageJ

For manuscripts utilizing custom algorithms or software that are central to the research but not yet described in published literature, software must be made available to editors/reviewers. We strongly encourage code deposition in a community repository (e.g. GitHub). See the Nature Research guidelines for submitting code & software for further information.

Data

Policy information about availability of data

All manuscripts must include a data availability statement. This statement should provide the following information, where applicable:

- Accession codes, unique identifiers, or web links for publicly available datasets
- A list of figures that have associated raw data
- A description of any restrictions on data availability

Source data for all figures will be provided with the paper. All other data supporting the findings of this study are available from the authors upon request.

Field-specific reporting

Please select the one below that is the best fit for your research. If you are not sure, read the appropriate sections before making your selection.

☒ Life sciences ☐ Behavioural & social sciences ☐ Ecological, evolutionary & environmental sciences

Life sciences study design

All studies must disclose on these points even when the disclosure is negative.

Sample size	Sample size was chosen based on prior experience of the investigators with similar experiments previously published. The authors have published numerous peer-reviewed papers demonstrating clear positive findings with similar sample sizes for the types of experiments included.
Data exclusions	No data points were excluded from the analysis of any of the experiments.
Replication	All experimental findings were reproduced in several independent experiments, as indicated in the figure legends.
Randomization	Randomization was performed by blinding investigators to genotype and allowing them to choose each subject blindly.
Blinding	Randomization was performed by blinding investigators to genotype and allowing them to choose each subject blindly. For studies using pharmacological agents, the investigator was aware of the agent being used, but was not aware of the genotypes of the animals used. Investigators were not aware of the specific group to which an animal was assigned to when doing the experiment or until after completion of the experiment.

Reporting for specific materials, systems and methods

We require information from authors about some types of materials, experimental systems and methods used in many studies. Here, indicate whether each material, system or method listed is relevant to your study. If you are not sure if a list item applies to your research, read the appropriate section before selecting a response.

Materials & experimental systems

n/a	Involved in the study	
☐	☒	Antibodies
☐	☒	Eukaryotic cell lines
☒	☐	Palaeontology
☐	☒	Animals and other organisms
☐	☒	Human research participants
☒	☐	Clinical data

Methods

n/a	Involved in the study	
☒	☐	ChIP-seq
☒	☐	Flow cytometry
☒	☐	MRI-based neuroimaging

Antibodies

Antibodies used

The following primary antibodies were used:
AMPKα antibody (Cell Signaling Technology #2532) (1:1,000)
AMPKβ antibody (Dr. Gregory Steinberg) (1:1,000)
Phospho-AMPKα (Thr172) (D4D6D) Rabbit mAb (Cell Signaling Technology #50081) (1:1,000)
AS160 (Abcam, #134749 (1:2,000)
Phospho-AS160 (Thr642) (D27E6) (Cell Signaling Technology) (1:1,000)
phospho-HSL (Ser565) (Cell Signaling Technology, #4137) (1:1,000)
Glut4 (1F8) (Cell Signaling Technology, #2213) (1:1,000)
Na,K-ATPase (Cell Signaling Technology, #3010) (1:1,000)
HA-tag (C29F4) (Cell Signaling Technology, #3724) (1:1,000)
β-actin (13E5) (Cell Signaling Technology, #4970) (1:1,000)

The following secondary antibodies were used:
Anti-mouse IgG, HRP-linked antibody (Cell Signaling Technology, #7076) (1:2,000)
Anti-rabbit IgG, HRP-linked antibody (Cell Signaling Technology, #7074) (1:2,000)

Validation

Validated by the manufacturer (for details, please visit the manufacturers' web sites)

Eukaryotic cell lines

Policy information about cell lines

Cell line source(s)	Mouse 3T3F442A cells (source: Kerafast); hWAT-SVF cells (source: Dr. Yu-Hua Tseng, Joslin Diabetes Center, Boston, MA)
Authentication	authenticated by Kerafast and Dr. Tseng's lab, respectively

Mycoplasma contamination	The cell lines tested negative for mycoplasma contamination.
Commonly misidentified lines (See ICLAC register)	No commonly misidentified cell lines were used in the study.

Animals and other organisms

Policy information about studies involving animals; ARRIVE guidelines recommended for reporting animal research

Laboratory animals	Male mice (C57BL6 background) older than 8 weeks; see the Methods section for detailed housing conditions
Wild animals	No wild animals were used in the study.
Field-collected samples	No field collected samples were used in the study.
Ethics oversight	All animal studies were approved by the NIDDK institutional Animal Care and Use Committee.

Note that full information on the approval of the study protocol must also be provided in the manuscript.

Human research participants

Policy information about studies involving human research participants

Population characteristics	For details, see ref:<br>Pydi SP, et al. Nat Commun. 2019 Jul 3;10(1):2936. doi: 10.1038/s41467-019-11003-4.
Recruitment	For details, see ref:<br>Pydi SP, et al. Nat Commun. 2019 Jul 3;10(1):2936. doi: 10.1038/s41467-019-11003-4.
Ethics oversight	We complied with all relevant ethical regulations for work with human participants. Subjects were admitted to the Metabolic Clinical Research Unit in the Hatfield Clinical Research Center of the National Institutes of Health (Bethesda, MD) to participate in an NIDDK/NIAMS institutional review board-approved protocol (ClinicalTrials.gov identifier NCT00428987), after having given informed consent.

Note that full information on the approval of the study protocol must also be provided in the manuscript.
